# Unraveling the Role of Particle Size and Nanostructuring on the Oxygen Evolution Activity of Fe-Doped NiO

**DOI:** 10.1021/acscatal.4c02329

**Published:** 2024-07-17

**Authors:** Reshma R. Rao, Alberto Bucci, Sacha Corby, Benjamin Moss, Caiwu Liang, Aswin Gopakumar, Ifan E. L. Stephens, Julio Lloret-Fillol, James R. Durrant

**Affiliations:** † Department of Materials, Royal School of Mines, Imperial College London, South Kensington Campus, London SW7 2AZ, U.K.; ‡ Grantham Institute—Centre for Climate Change and the Environment, Imperial College London, South Kensington Campus, London SW7 2AZ, U.K.; § Institute of Chemical Research of Catalonia (ICIQ), The Barcelona Institute of Science and Technology, Avinguda Països Catalans 16, Tarragona 43007, Spain; ∥ Department of Chemistry, Centre for Processable Electronics, Imperial College London, London W12 0BZ, U.K.; ⊥ Catalan Institution for Research and Advanced Studies (ICREA), Passeig Lluıs Companys, 23, Barcelona 08010, Spain

**Keywords:** water oxidation, catalysis, spectroelectrochemistry, mechanism, kinetics, nickel oxide

## Abstract

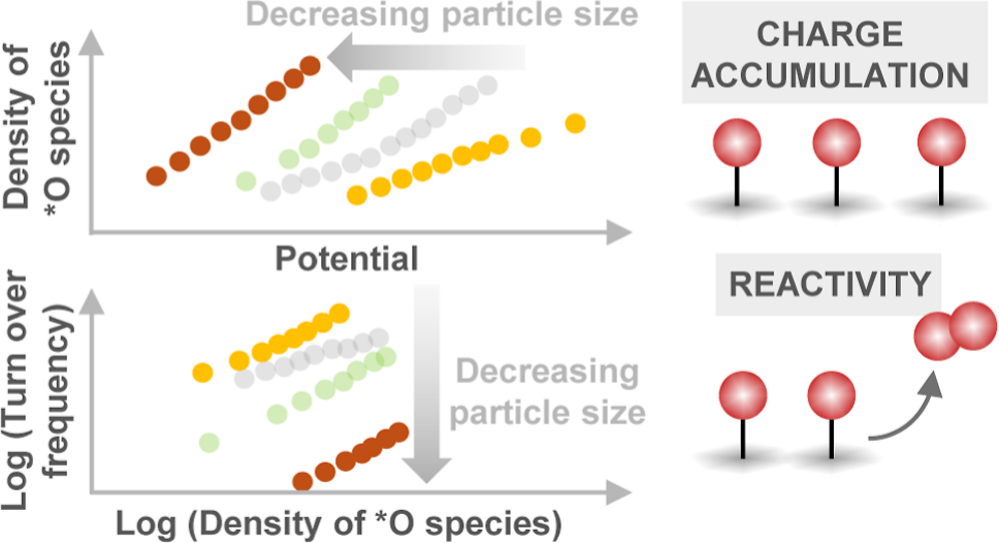

Nickel-based oxides and oxyhydroxide catalysts exhibit state-of-the-art activity for the sluggish oxygen evolution reaction (OER) under alkaline conditions. A widely employed strategy to increase the gravimetric activity of the catalyst is to increase the active surface area via nanostructuring or decrease the particle size. However, the fundamental understanding about how tuning these parameters influences the density of oxidized species and their reaction kinetics remains unclear. Here, we use solution combustion synthesis, a low-cost and scalable approach, to synthesize a series of Fe_0.1_Ni_0.9_O samples from different precursor salts. Based on the precursor salt, the nanoparticle size can be changed significantly from ∼2.5 to ∼37 nm. The OER activity at pH 13 trends inversely with the particle size. Using operando time-resolved optical spectroscopy, we quantify the density of oxidized species as a function of potential and demonstrate that the OER kinetics exhibits a second-order dependence on the density of these species, suggesting that the OER mechanism relies on O–O coupling between neighboring oxidized species. With the decreasing particle size, the density of species accumulated is found to increase, and their intrinsic reactivity for the OER is found to decrease, attributed to the stronger binding of *O species (i.e., a cathodic shift of species energetics). This signifies that the high apparent OER activity per geometric area of the smaller nanoparticles is driven by their ability to accumulate a larger density of oxidized species. This study not only experimentally disentangles the influence of the density of oxidized species and intrinsic kinetics on the overall rate of the OER but also highlights the importance of tuning these parameters independently to develop more active OER catalysts.

## 1 Introduction

The oxygen evolution reaction (OER) is one of the most prominent anodic reactions for aqueous electrochemical energy conversion and storage technologies since it can be coupled with the hydrogen evolution reaction^1^ or the reduction of carbon dioxide to energy-dense liquid fuels^2^ at the cathode. In alkaline conditions, nickel-based oxides^3^ and hydroxides^4−6^ exhibit state-of-the-art activity. However, the sluggish kinetics of this reaction still necessitates the application of an overpotential to achieve industrially relevant current densities, thus directly reducing the efficiencies of these technologies.

Since electrochemical reactions are typically confined to the surface, increasing the surface area via nanostructuring or decreasing the particle size is a widely employed strategy to increase the activity^7−9^ per geometric area of the electrode or mass of the catalyst. Solution combustion is a cost-effective synthesis route to produce metal oxide nanoparticles with chemical and structural tunabilities.^10,11^ By combusting metal salts, an oxidant, and a fuel in appropriate ratios, self-combustion can be triggered with copious gas evolution, resulting in foamy and porous oxides with a large surface area. In our recent work, we have demonstrated the applicability of solution combustion synthesis, using nickel nitrate and iron chloride salts as precursors, for synthesizing self-supported Fe-doped NiO catalysts on Ni foam.^12^ The resultant catalysts had a water oxidation activity of 10 mA/cm^2^_geometric_ at an overpotential of 190 mV.^12^ Altering the precursor metal salts and the ratio of the fuel to oxidants in the starting solution can significantly alter the combustion properties and tune the size and morphology of the resultant nanoparticles.^13−17^ For example, LiMn_2_O_4_ particles prepared with varying ratios of NO_3_^–^/CH_3_COO^–^ precursor salts produced significantly different nanocrystallite sizes. When used as Li-ion battery cathode materials, samples obtained with the optimal 3:2 ratio of NO_3_^–^/CH_3_COO^–^ salts demonstrated the best rate capability, highest charge–discharge capacity, and lowest voltage hysteresis, owing to their smallest nanocrystallites and primary particle sizes.^13^ However, the effect of synthesis parameters on the nanostructure of water oxidation catalysts and, consequently, its effect on the activity remains unknown.

Understanding how tuning the morphology and particle size of catalysts alters the water oxidation kinetics is challenging since nanostructuring can influence the density of redox-active species and their intrinsic kinetics. For example, a study on size-selected NiFe nanoparticles has shown that although the gravimetric activity depends on the particle diameter (particles with 6.7 nm diameter exhibit a mass activity of ∼35 A/mg_NiFe_, whereas smaller particles with 3.9 nm diameter have a much higher activity of ∼110 A/mg_NiFe_ at 1.60 V_RHE_),^18^ when normalized to the surface area, the activity is comparable. On the contrary, a recent study on size-selected CoOOH nanoparticles has shown that the intrinsic activity increases for sub-5 nm size nanoparticles. The activity normalized to the surface area for 1 nm nanoparticles is ∼2.5 times that of 4 nm size nanoparticles. This increase in activity can be correlated to the smaller increase in the accumulation of oxidation charge and Co–O contraction during OER compared to the ideal value calculated based on the surface area-to-volume ratio changes.^19^ Recent mechanistic studies using a combination of spectroscopy and density functional theory calculations on iridium,^20−25^ nickel,^26,27^ and cobalt-based catalysts^28^ have demonstrated the crucial role of cooperative effects between adjacent oxidized centers in enabling the rate-determining O–O bond formation step. Consequently, the energetics of O–O bond formation might also be affected by a change in the accumulation of oxidized species and the strength of the metal–oxygen bond, which can vary depending on the particle size. However, the impact of morphology and particle size on these interactions between oxidized states and thus the oxygen evolution kinetics remains unknown.

Experimentally, it is challenging to understand whether differences in gravimetric current densities for nanostructured catalysts arise from differences in the density of active species or their intrinsic water oxidation kinetics. This fundamental insight requires experimentally probing the number of redox-active species, which is not trivial, particularly for systems that are less well-defined. For Ni-based electrocatalysts, the density of active Ni centers is often determined by the area of the Ni^2+^/Ni^3+^ redox transition prior to oxygen evolution. However, an earlier paper from our laboratory has shown, using spectroelectrochemistry on NiO samples with different thicknesses, that the Ni^2+^/Ni^3+^ redox transition at ∼1.45 V_RHE_ includes oxidation of bulk Ni centers, whereas oxidized species formed at water oxidation-relevant potentials are present only on the surface.^29^ The absence of a correlation between the electrochemically measured charge passed in the Ni^2+^/Ni^3+^ redox and the number of oxidized species formed at water oxidation potentials has also been observed on doped NiO nanoparticles^26^ and Ni_*x*_Fe_1–*x*_OOH particles,^27^ thus questioning the validity of the redox peak area as an indicator for the density of redox-active species for these oxides. Therefore, these uncertainties in determining the density of redox-active species under the reaction conditions make the deconvolution of intrinsic and extrinsic effects on the electrocatalytic activity challenging.

Our previous studies established solution combustion as a suitable method for synthesizing catalytic metal oxides with enhanced activity.^12,26^ This report introduces another dimension of tuning the catalyst properties by combining sol–gel chemistry with solution combustion. Starting from our previously developed NiO catalyst with a 10% Fe dopant as a model system, we attempted to control the level of nanostructuring and particle size through the sol–gel kinetics of NiFe seed nucleation. In particular, we hypothesized that the heat of combustion could directly influence the size of the nanoparticles formed, and, consequently, we explored different metal precursor salts (nitrate, chloride, acetylacetonate, and sulfate) to synthesize them. We show how the precursor salts influence the heat of combustion and consequently the particle size; the nitrate-derived particles are ∼37 nm in diameter, while the sulfate-derived particles are ∼2.5 nm in size. At pH 13, the smaller particles exhibit higher water oxidation activity normalized to not only the geometric area but also the particle surface area of the catalyst. Using time-resolved spectroelectrochemistry, we quantitatively detect the density of oxidized Ni species at water oxidation-relevant potentials. The water oxidation current demonstrates second-order dependence on the density of oxidized Ni species, suggesting that O–O coupling between two adjacent oxidized Ni centers is the rate-determining step of the reaction in the potential regime of measurement. We find that the smallest sulfate-derived sample accumulates a significantly larger number of oxidized species per oxide surface area at a given potential. However, the kinetics per oxidized state is the slowest. Thus, we rationalize that the increasing activity per oxide surface area is driven by the ability of the small particles to accumulate oxidized species, although the reactivity of these species is slow. This finding provides new insights into the design of nanostructured materials that increase the density of redox-active species, which results in a higher current density for water oxidation.

## 2 Results and Discussion

### 2.1 Material Synthesis and Characterization

**Figure 1:**
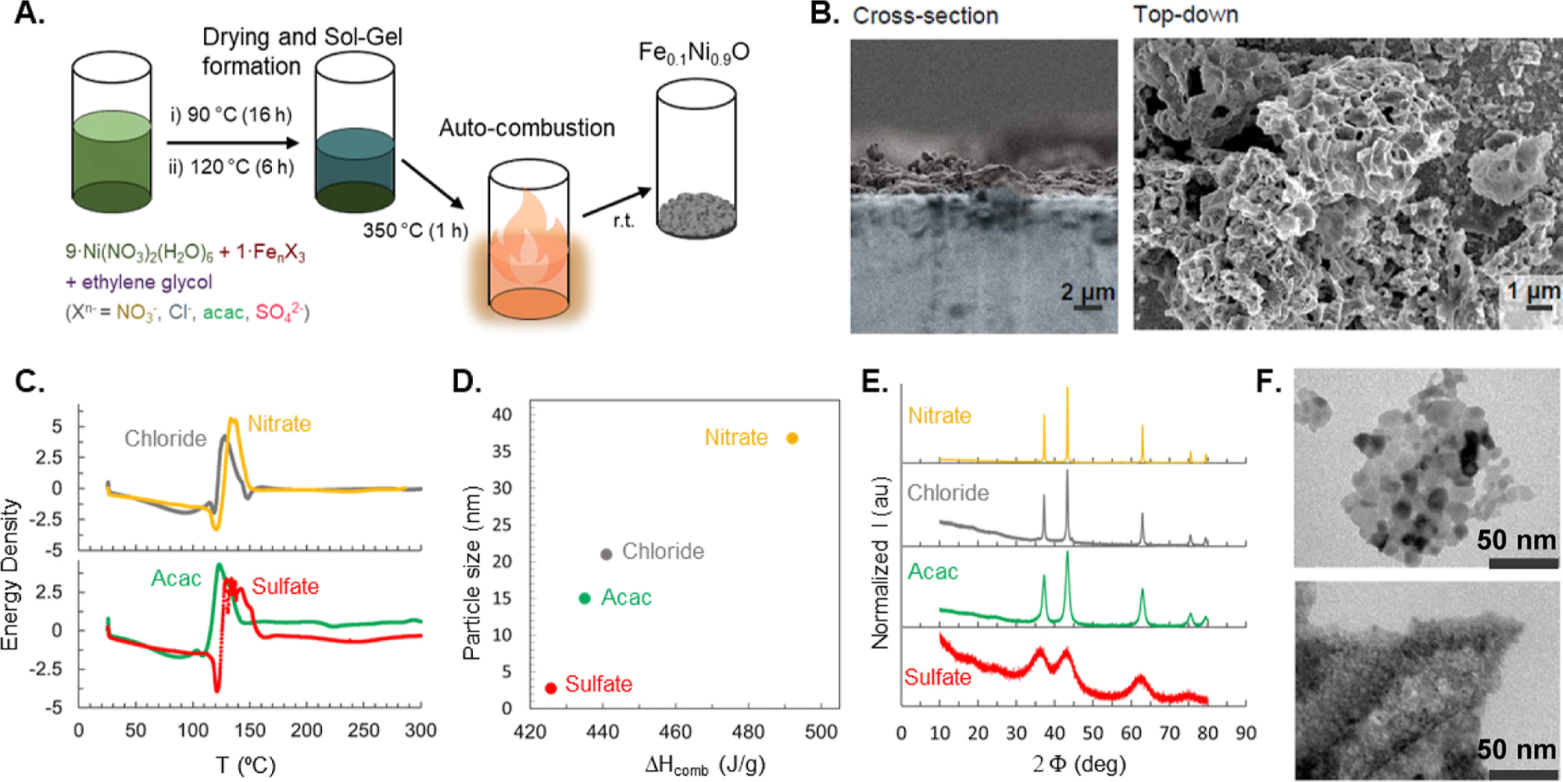
(A) Scheme of the solution combustion synthesis process. Ni(NO_3_)_2_(H_2_O)_6_ was used as the Ni source, Fe_*n*_X_3_ (X^*n*–^ = Cl^–^, NO_3_^–^, CH_3_COCHCOCH_3_^–^ (acac), SO_4_^2–^) salts were used as the Fe source, and ethylene glycol was used as the coordinating agent and fuel. The resultant nanoparticles were spray-coated on an FTO substrate. (B) Cross section and top-down scanning electron microscopy (SEM) images of a nitrate-derived Fe_0.1_Ni_0.9_O showing the resultant foamy morphology. (C) Energy density as a function of temperature for the nitrate-, chloride-, acac-, and sulfate-derived nanoparticles. (D) Particle size of as-synthesized nanoparticles as a function of combustion enthalpy. (E) XRD patterns of the nitrate-, chloride-, acac-, and sulfate-derived samples showing the presence of the rock salt phase. (F) Transmission electron microscopy (TEM) images of the nitrate- (top) and sulfate- (bottom) derived nanoparticles.

Fe_0.1_Ni_0.9_O samples were synthesized using solution combustion, as shown in Figure 1A using different precursor salts. A reactive mixture of nickel nitrate, an iron salt (chloride, nitrate, acetylacetonate, and sulfate), and ethylene glycol fuel was heated at an oxidant-to-fuel ratio of 1. The pH value of the solution was initially adjusted to pH 1. After homogenization, the pH was adjusted to 7 to start the sol–gel formation at 90 °C overnight. This phase of the reaction is necessary to start the nucleation and growth of the nanoparticle’s seeds. The nucleation and growth processes start in the sol–gel phase of the reaction before the ignition temperature. This initial step can be simplified considering the condensation reactions of M^2+^ in the M(O)M species as follows.
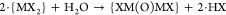
When forming the Ni(O)Ni species, the size of the nucleated seeds increases, as well as the solution pH. Consequently, the sol–gel process growth is controlled not only by pH but also by ions coordinated to the metal, which controls the hydrolysis and condensation processes. Indeed, in our case, during the aging process at 90 °C, the final solution pH increases in the following order: nitrate < chloride < sulfate < acac, with pH values of 1.5, 2, 3, and 4.5, respectively. As judged by the pH values, we assume that the size of the seeds should be the highest for the nitrate and the lowest for acac. For comparison purposes, an undoped NiO sample was also prepared from Ni(NO_3_)_2_(H_2_O)_6_ following the same procedure.

Then, after the sol–gel was dried at 120 °C for 6 h, a fast shock exothermic reaction is triggered by increasing the temperature in the muffle furnace due to the reaction between the nitrate NO_3_^–^ and ethylene glycol to form NiO, Ni(NO_3_)_2_(H_2_O)_6_ + C_2_H_6_O_2_ → NiO + 2CO_2_ + 9H_2_O + N_2_. The nickel nitrate and iron salts are included in a 9:1 ratio to ensure that the synthesized oxide has 10% Fe doping, as confirmed by inductively coupled mass spectrometry (Table S1). The resultant nanoparticles are spray-coated on FTO substrates with a loading of 2.5 mg/cm^2^_geometric_ for further testing. In the following discussion, the samples have been denoted as nitrate, chloride, acac, and sulfate, describing the anion of the iron salt used in the synthesis. The vigorous gas evolution during the process results in oxides with a foamy morphology, as seen in the SEM images in Figures 1B and S1. The energy density during the solution combustion process was obtained using differential scanning calorimetry as a function of temperature, Figure 1C. The total heat of the reaction during the synthesis, i.e., the combustion enthalpy Δ*H*_comb_, is largest in the nitrate-derived sample and smallest in the case of the sulfate-derived sample. The chemical composition of the fuel and oxidizer, fuel-to-oxidizer ratio, gas atmosphere, solution pH, and combustion temperature are known to affect the heat of combustion and the resultant microstructure.^10^ It is interesting to note that the pH value obtained after aging follows the combustion enthalpy trend, except for the acac ligand. However, the acac ligand can also be seen as a fuel like ethylene glycol, and this could explain the higher enthalpy than that in the case of sulfate.

The particle size of Fe_0.1_Ni_0.9_O nanoparticles scales with the heat of the reaction. The nanoparticle size can be extracted from the powder X-ray diffraction (PXRD) (Figure 1E) as well as the TEM images (Figures 1F and S2–S10). The prepared NiO nanoparticles exhibit a nonuniform particle distribution, with diameters ranging from ∼10 to ∼30 nm, and demonstrate a highly crystalline nature, as evidenced by distinct spots and rings in the fast Fourier transform (FFT) diffractograms (Figure S2). Additionally, the selected-area electron diffraction (SAED) pattern reveals distinct rings that closely match the simulated reference for NiO cubic crystals (Figure S3). STEM-EDS images confirm the localized presence of Ni and O within the sample (Figure S4). Samples derived from sulfate show the smallest particle size of about 2.5 nm, whereas those derived from nitrate display the largest particle size, ∼37 nm (Figures S5,S6,S9, and S10). The crystallinity of the particles is further confirmed by distinct spots and rings in the FFT diffractograms (Figure S6) and the ring pattern in SAED (Figure S7). In the XRD pattern, peak broadening in acac- and sulfate-derived samples indicates a reduction in the crystallite size. This broadening aligns with the diffused nature of rings in the SAED pattern (Figure S7), which suggests a very small particle size. Moreover, the STEM-EDS images confirm the localized presence of Ni, Fe, and O within the sample (Figure S8). Although the particle sizes are different, all oxides are present in the rock salt phase. While bulk XRD and previous X-ray absorption measurements suggest the presence of rock salt NiO in the as-prepared samples, the surface of all samples is enriched with Ni^3+^, as seen in the X-ray photoelectron spectra (Figure S11), which can be attributed to the presence of surface defects caused by the formation of cationic vacancies.^12^ The presence of surface defects is also supported by Raman spectra reported in our previous work,^26^ which show bands at ∼500 cm^–1^ that are only present in disordered and defect-rich samples and cannot be observed in perfect NiO single crystals. Thus, using a facile approach, we demonstrate how a range of different nanoparticle sizes can be obtained for the same chemical composition.

**Figure 2:**
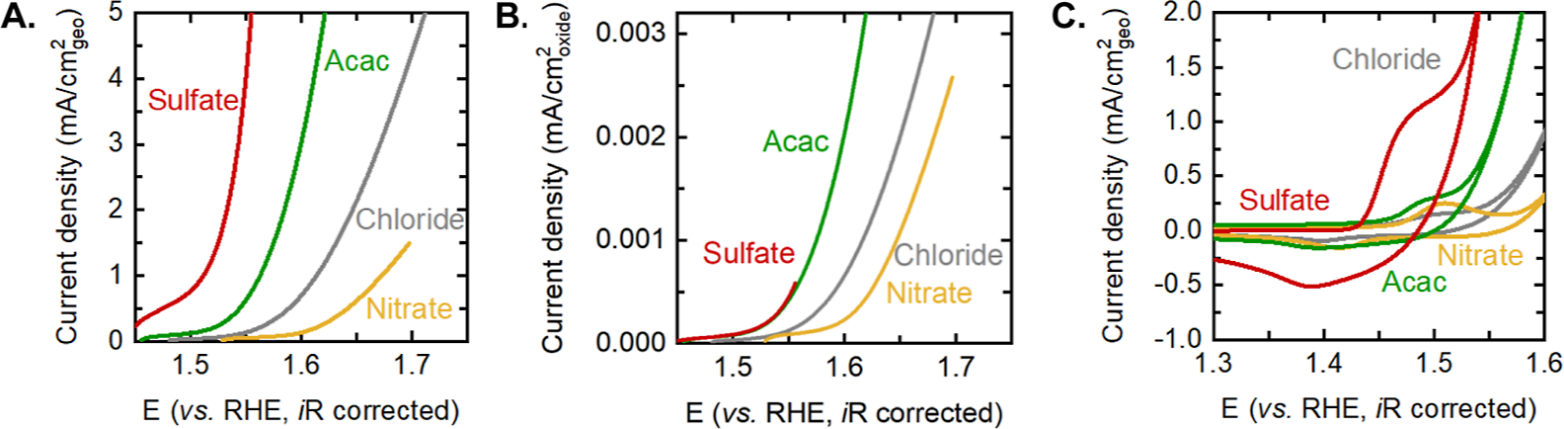
(A) Linear sweep voltammograms of the nitrate-, chloride-, acac-, and sulfate-derived Fe_0.1_Ni_0.9_O. (B) Linear sweep voltammograms of the nitrate-, chloride-, acac-, and sulfate-derived Fe_0.1_Ni_0.9_O normalized to the oxide surface area. (C) Cyclic voltammograms of the nitrate-, chloride-, acac-, and sulfate-derived Fe_0.1_Ni_0.9_O. The measurements were made on samples deposited on FTO at a scan rate of 10 mV/s in 0.1 M Fe-free KOH. Pt was used as the working electrode, and Ag/AgCl was used as the counter electrode. The oxide surface area was calculated by using an estimate of the particle diameter from the PXRD and TEM measurements.

The electrochemical activity normalized to the geometric area of the electrode measured in Fe-free 0.1 M KOH scales with the particle size, being the largest for the sulfate-derived samples and the smallest for the nitrate-derived samples, as shown in Figure 2A. The linear sweep voltammograms were measured after the films were cycled 50 times between 1.2 V_RHE_ and an anodic potential corresponding to ∼1 mA/cm^2^_geometric_. The current density measured on the smallest sulfate-derived samples is 2 orders of magnitude larger than that measured on the nitrate-derived samples. Notably, the best-performing sulfate-derived samples are able to achieve 30-fold improvement in activity relative to the state-of-the-art chloride-derived Fe_0.1_Ni_0.9_O reported in our previous work.^12^

Based on literature reports on phosphide-,^30,31^ nitride-,^32,33^ carbide-,^34^ and chalcogenide-based^35,36^ catalysts, it can be seen that complete leaching of the anion and conversion to the oxyhydroxide phase occur on the surface of the catalyst after exposure to OER potentials. Therefore, the chemical nature of the anion alone cannot account for the change in the reactivity of surface Ni sites. Differences in surface area alone are also not enough to account for the differences in activity, as shown by the surface area-normalized activity in Figure 2B. The oxide area was obtained using the estimate of the particle diameter, *d*, from PXRD and TEM, via the following equation: , where ρ is the density of the oxide and *m* is the mass loading.^7^ Interestingly, we observed that even after accounting for differences in the surface area, the sulfate- and acac-derived samples have ∼3 and ∼8 times the activity of the chloride- and nitrate-derived samples, respectively. The differences in the surface area-normalized current density can stem from the difference in the number of redox-active centers per geometric area of the catalyst and/or from the difference in the intrinsic activity per redox-active center. In order to untangle the contribution from these two effects and obtain a mechanistic understanding of the differences in the electrochemical activity of these samples, we next use time-resolved operando spectroscopy to determine the potential-dependent density of oxidized species and their kinetics for water oxidation.

### 2.2 Quantifying the Density of Oxidized Species and Their Redox Kinetics Using Operando Spectroelectrochemistry

On increasing the potential from 1.2 V_RHE_ to water oxidation conditions, a prominent redox transition was observed for all the samples in the cyclic voltammogram at ∼1.45 V_RHE_ (Figures 2C and S12). This redox peak was assigned to the Ni^2+^/Ni^3+^ redox transition via the following redox reaction: Ni(OH)OH + OH^–^ → Ni(OH)O + H_2_O + e^–^.^26,37,38^ The average peak position of the redox center is weakly dependent on the precursor salt, being ∼1.45 V_RHE_ for the nitrate-, chloride-, and acac-derived samples and only showing a noticeable change to ∼1.42 V_RHE_ for the sulfate-derived samples. The relatively small change in the redox peak position suggests that the binding strength of oxygenated species on the Ni center is weakly influenced by the synthesis route, compared to the changing nature and composition of the doped cation observed in previous studies.^26^ The charge passed during the redox transition increases with a decrease in the particle size, indicating that smaller particles have larger electrochemically active sites participating in the redox transition per geometric area due to the larger surface-to-volume ratio of the particles. However, the charge passed for the smallest sulfate-derived particles is only 3.5 times that for the largest nitrate-derived particles, significantly smaller than the 15 times increase in surface area of the nanoparticles and ∼8 times increase in specific area-normalized activity. This is consistent with a recent work on CoO_*x*_(OH)_*y*_ catalysts, where an increase in the accumulation of oxidative charge was found with the decreasing particle size, but the degree of this increase was lesser than that predicted by only considering the surface-to-volume ratio.^19^ It has been reported that additional effects such as the degree of oxidation of Ni sites and preferential surface orientation can also contribute to differences in the redox charge.^7^ Therefore, while the greater charge in the case of the sulfate-derived samples is indicative of a larger density of redox-active sites, this analysis cannot be used to accurately quantify the density of the electroactive species. Indeed, previous studies have shown no correlation between the charge passed during this redox transition and the density of active states probed during water oxidation conditions.^27,29^

Upon increasing the potential to the water oxidation region, a second redox transition following that observed in the cyclic voltammogram at ∼1.45 V_RHE_ (Figure 2C) was detected by using optical spectroscopy (Figure S13). At water oxidation-relevant potentials, an increase in absorption at ∼500, ∼750, ∼650, and ∼850 nm was observed for the chloride-, nitrate-, acac-, and sulfate-derived samples, respectively, Figure S14. Interestingly, although the maximum wavelength of absorption was different between these samples, it was found to be similar between Fe_0.1_Ni_0.9_O and Zn_0.1_Ni_0.9_O samples prepared by using the same salt (Figure S15), suggesting that the wavelength of absorption was dependent on the anion used in the synthesis, which could alter the local environment of the active Ni site. This absorption has been assigned to d–d interband transitions^39,40^ and corresponds to changes in optical properties stemming from the further oxidation of Ni centers. The oxidation of Ni has been proposed to be coupled with the deprotonation of *OH groups to form *O via Ni(OH)O + OH^–^ → NiOO + H_2_O + e^–^,^38,40,41^ where these oxo species can have a negative charge.^38,42^ Therefore, an increase in optical absorption can be directly related to the formation of *O species.

**Figure 3:**
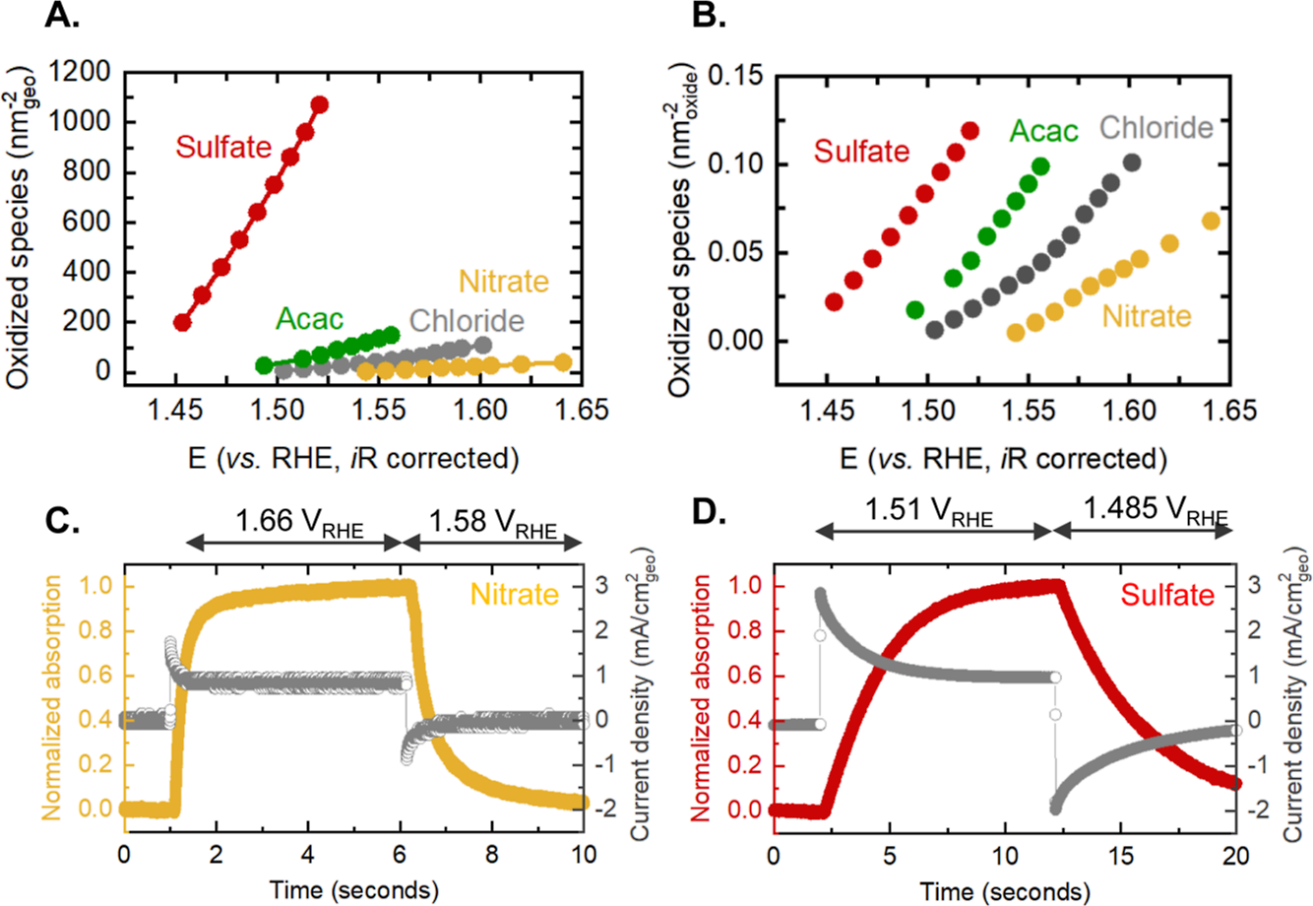
Density of oxidized Ni^4+^–*O species obtained using operando UV vis spectroelectrochemistry for the nitrate-, chloride-, acac-, and sulfate-derived Fe_0.1_Ni_0.9_O normalized to (A) geometric surface area and (B) oxide surface area. Stepped potential spectroelectrochemistry for the (C) nitrate-derived samples, with a potential of 1.66 V_RHE_ applied from 1 to 6 s, followed by a potential of 1.58 V_RHE_ applied from 6 to 10 s, probed at a wavelength of 750 nm and (D) sulfate-derived samples, with a potential of 1.51 V_RHE_ applied from 2 to 12 s, followed by a potential of 1.485 V_RHE_ applied from 12 to 20 s, probed at a wavelength of 850 nm. The potential windows are chosen to be in regions where the second redox transition of the Ni centers is detected. The left *y-*axis depicts the normalized absorption, and the right *y-*axis shows the current density. The measurements were made on samples deposited on FTO in 0.1 M Fe-free KOH. Pt was used as the working electrode, and Ag/AgCl was used as the counter electrode. The applied potential is corrected for *iR* compensation. The oxide surface area was calculated using an estimate of the particle diameter from the PXRD and TEM measurements.

A quantitative analysis of the density of oxidized species corresponding to the increase in absorption was performed by complementary stepped potential spectroelectrochemistry, as described in our previous work^22,23,26,27^(Figures S16–S19). In short, a potential step was applied in a potential regime where these oxidized species form. On applying a step increase in the potential, the absorption at the characteristic wavelength for each sample was found to increase, and upon returning to the original potential, the absorption decreases. The charge passed during the reduction can be correlated to optical intensity for a series of such stepped potential measurements to extract the extinction coefficient and consequently the potential-dependent density of oxidized species. As can be seen from Figure 3A, the accumulated charge at a given potential increases with decreasing particle size, being the maximum for the sulfate-derived nanoparticles. The density of these oxidized species normalized to the oxide surface area of the particles shows that at a given potential, the number of oxidized species per oxide surface area increases with decreasing particle size (Figure 3B). The extrapolated onset for the formation of these oxidized species also follows a similar trend of the redox peak observed in the cyclic voltammogram, being the lowest for the smallest sulfate-derived samples, ∼1.44 V_RHE_, and increasing to ∼1.48 V_RHE_, ∼1.51, and ∼1.52 V_RHE_ for the acac-, chloride-, and nitrate-derived samples. This cathodic shift in the formation of oxidized species is indicative of stronger binding of *O. Based on our previous work correlating the binding energetics to reaction kinetics on a number of cation-doped NiO samples, a stronger binding energy of *O intermediates relative to the chloride-derived Fe_0.1_Ni_0.9_O (i.e. cathodically shifted redox potential) should result in a decrease in intrinsic water oxidation kinetics.^26^ We note that for the chloride-derived sample, a kink in the density of oxidized species as a function of potential has been observed at 1.55 V. This nonlinear dependence can be attributed to the interaction between adsorbates formed on the surface at these potentials, which alter their binding energetics and consequently the potential dependence for their formation. Such a behavior has recently been demonstrated for NiOOH,^27^ CoOOH^28^, and IrO_*x*_^20−25^ electrodes.

The time constant for the oxidation/reduction of these species also depends on the particle size, as shown in Figure 3C,D. For the largest nitrate-derived samples, the time constant of oxidation/reduction obtained from the time-dependent increase/decrease of the optical signal is ∼0.45 s. On the other hand, for the smallest sulfate-derived samples, this time constant is approximately an order of magnitude longer, ∼3.4 s (Figures S20 and S21). We note that these time constants are independent of the magnitude of the potential step, as shown in Figures S20 and S21, and are measured in a potential regime where significant water oxidation does not occur. These time constants can thus be attributed to the oxidation of Ni centers from *OH to *O rather than to the kinetics of water oxidation. Similar measurements on Ni_*x*_Fe_1–*x*_ nanoparticles have reported a time constant of ∼0.06 s for *x* = 0.86 and ∼0.8 s for *x* = 0.27, with the larger time constant for higher Fe doping being attributed to the higher charge-transfer kinetics.^41^ In this case, all four materials have the same catalyst composition. We thus attribute the difference in time constant for oxidation–reduction to the difference in charge transport between the samples. We hypothesize that the slower charge transport on the smallest sulfate-derived samples is due to the larger number of grain boundaries and consequently a higher contact resistance between particles. Therefore, based on the time-resolved tracking of the oxidation of Ni centers, we can not only determine the density of these redox centers but also compare the time constant of these processes.

### 2.3 Intrinsic Water Oxidation Kinetics

**Figure 4:**
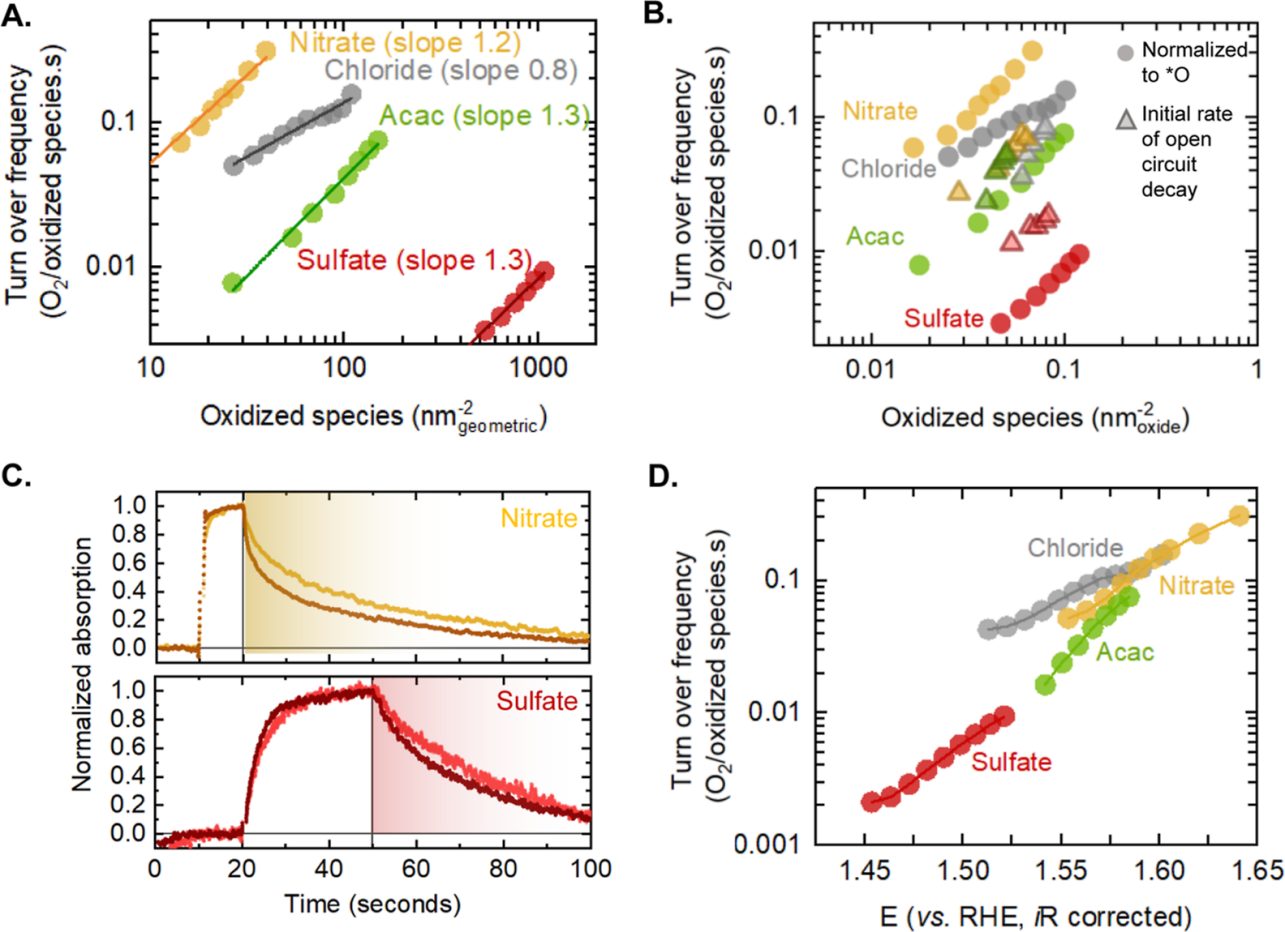
(A) Turnover frequency obtained by normalizing the current density to the density of oxidized species as a function of the number of oxidized species per geometric area for the nitrate-, chloride-, acac-, and sulfate-derived samples. Faradaic efficiency of oxygen production has been assumed to be 100%. (B) Turnover frequency obtained by normalizing the current density to the density of oxidized species (circles) as a function of the number of oxidized species per oxide surface area. Turnover frequency determined using the initial rate method of the open-circuit decay has been shown as triangles of the corresponding color. (C) (top) Open-circuit decay measurements for the nitrate-derived sample from 1.57 V_RHE_ (∼0.028 species/nm^2^_oxide_), yellow, and 1.63 V_RHE_ (∼0.06 species/nm^2^_oxide_), brown. The sample is held at a constant potential from 10 to 20 s, followed by open-circuit decay from 20 to 100 s. (bottom) Open-circuit decay measurements for the sulfate-derived sample from 1.47 V_RHE_ (∼0.05 species/nm^2^_oxide_), pink, and 1.495 V_RHE_ (∼0.074 species/nm^2^_oxide_), dark red. The sample is held at a constant potential from 20 to 50 s, followed by open-circuit decay from 50 to 100 s. The measurements were made on samples deposited on FTO in 0.1 M Fe-free KOH. Pt was used as the working electrode, and Ag/AgCl was used as the counter electrode. The applied potential is corrected for *iR* compensation. (D) Turnover frequency as a function of potential for the nitrate-, chloride-, acac-, and sulfate-derived samples. The oxide surface area was calculated using an estimate of the particle diameter from the PXRD and TEM measurements.

Next, we correlate the turnover frequency (defined as the amount of oxygen produced, normalized to the density of oxidized species per second) with the density of oxidized species. The kinetics of water oxidation on a range of (photo)electrodes has been shown to depend on the density of oxidized species, with the role of applied potential being solely to increase the density of these oxidized species.^21,26,27,43−45^ Our previous work has shown that, specifically for nickel-based oxides and oxyhydroxides,^26,27^ within the potential regime measured, the current can be related to the density of oxidized species using the rate equation: *J* = *k* × [oxidized species]^α^, where *J* is the current density, *k* is the potential-independent water oxidation rate constant, and α is the order of the reaction with respect to the density of oxidized species. Consequently, the turnover frequency, defined as the oxygen evolved per oxidized species, can be written as TOF . Figure 4A shows a log–log plot of the TOF versus the density of oxidized species; the equivalent plot of current density versus oxidized species is shown in Figure S22. A straight line with a slope of ∼1 is obtained for all the samples, suggesting that the reaction rate is second order with respect to the density of oxidized species, i.e., α = 2. From this analysis, we can conclude that the rate-determining step involves the chemical combination of two adjacent oxidized Ni species (Ni^4+^–*O) to form molecular oxygen. This description is consistent with our previous work on cation-doped NiO^26^ as well as previous mechanisms for NiB_*i*_,^46^ CoP_*i,*_^47,48^ and CoOOH.^49,50^ We note that the kinetic analysis is based on data collected over 1 order of magnitude of current density (Figure S22). A larger range of current densities provides information about the applicability of this kinetic regime at lower and higher potentials. However, the analysis at low overpotentials is currently limited by the low faradaic efficiency of OER in this regime,^51^ and the measurements at high potentials are challenging due to the distortion of the optical signal by bubble accumulation. Advances in electrochemical mass spectrometry to measure oxygen at low potentials^51^ and time resolution of optical spectroscopy to acquire data at high potentials without significant bubble buildup may help address these challenges.

Although all of the materials exhibit approximately equivalent reaction order and are thus likely to follow the same reaction mechanism, the turnover frequency per surface area-normalized oxidized species is strongly dependent on the particle size. For the smallest sulfate-derived nanoparticles, the turnover frequency is 1 order of magnitude smaller than the other samples. This trend of decreasing turnover frequency with particle size is further validated by comparing these results to those obtained by using open-circuit decay measurements. The open-circuit decay measurement technique was initially used by Conway et al. in 1950s, where it was shown that the potential decay from water oxidation potentials to the open circuit was accompanied by the evolution of oxygen.^52^ With our time-resolved UV–vis spectroscopy setup, we can track the optical signal change during the potential decay with ∼30 ms time resolution. The rate of initial decay obtained using this measurement technique provides insight into the turnover time for a given density of species present before the onset of the decay. Figure 4C shows normalized decay spectra for the nitrate- and sulfate-derived samples, and Figure S23 shows the data for the chloride- and acac-derived samples. For the nitrate-derived sample, the potential is held at 1.57 V_RHE_ (yellow curve) and 1.63 V_RHE_ (brown curve) between 10 and 20 s corresponding to ∼0.028 and ∼0.063 species/nm^2^_oxide_, respectively. At 20 s, the cell is switched to the open-circuit decay mode. The initial decay time changes from ∼9 s at a low potential to ∼3.6 s at a high potential, equivalent to turnover frequencies of 0.028 and 0.07 O_2_/oxidized species per second, respectively. For the sulfate-derived sample, the potential was held at 1.47 and 1.495 V_RHE_ between 20 and 50 s, corresponding to ∼0.05 and ∼0.07 species/nm^2^_oxide_, respectively. We note that a longer time was chosen compared with the other samples to account for the significantly slower nickel oxidation kinetics. The fast component of the decay varied from ∼22 to ∼12 s. The resulting turnover frequencies (0.011 and 0.02 O_2_/oxidized species per second, respectively) are significantly slower than the nitrate-derived sample. Therefore, although the smaller nanoparticles can accumulate a larger density of oxidized species, the intrinsic activity of these oxidized species is lower.

The lower intrinsic activity for smaller nanoparticles can be due to a number of effects. First, the stronger binding of the *O species (i.e., cathodic shift of species energetics) with a decreasing particle size results in them being less reactive. Our previous work on cationic-doped, chloride-derived NiO particles suggested that an optimal binding of *O species is required to ensure that the *O species are formed at low overpotentials. However, binding *O species stronger than optimal possibly results in the O–O formation and O_2_ removal step, limiting the overall rate of the reaction. Chloride-derived Fe_0.1_Ni_0.9_O was found to have near-optimum binding energetics.^26^ Consequently, for particles smaller than the chloride-derived samples, such as the sulfate-derived samples, the stronger *O binding can result in lower reaction kinetics owing to the slow kinetics of O–O formation and removal. Second, differences in the morphology and packing of the smaller nanoparticles have shown to impact the charge-transfer kinetics (Figure 3C,D) and could potentially also impact the kinetics of O–O bond formation due to larger spatial separation or steric effects between the neighboring oxidized states.

Finally, based on our results, we determine the potential dependence of the turnover frequency (Figure 4D). The turnover frequency at a given potential is a result of two competing effects—(1) the species accumulated, which scales inversely with the particle size and (2) the reactivity of each oxidized species, which scales directly with the particle size. For example, at 1.525 V_RHE_, the smallest sulfate-derived samples have ∼0.12 species/nm^2^_oxide_, while the chloride-derived samples only have ∼0.025 species/nm^2^_oxide_. Although the sulfate-derived nanoparticles have ∼6 times the density of oxidized species, the slower rate of coupling of these species to facilitate O–O bond formation on these particles results in lower turnover frequencies at 1.525 V_RHE_. Therefore, the higher geometric area-normalized activity of the small sulfate-derived particles observed in Figure 2A can be largely attributed to the accumulation of oxidized species.

Combining the spectroscopic results of the potential-dependent density of oxidized species and the intrinsic reaction kinetics provides important insights for the design of more active catalysts. Significant efforts over the past decades have been focused on nanostructuring catalysts to increase the effective surface area. However, recent advances in mechanistic insight of the mechanism of water oxidation on metal oxides^20−23^ and oxyhydroxides^26,27^ suggest cooperative effects between oxidized centers can improve the OER activity. Therefore, the design of nanostructured materials should consider not only increasing the number of redox-active species per geometric area of the electrode but also facilitating their interaction. The optimal catalyst would be able to accumulate the same density of oxidized states as observed in the smaller nanoparticles but achieve the reaction kinetics of the larger nanoparticles, which could further improve the OER activity by ∼1 to 2 orders of magnitude. In this regard, solution combustion synthesis offers a low-cost and scalable platform for tailoring nanostructures by tuning the precursor salts to achieve catalyst particles with a range of morphologies and nanostructures to achieve the optimum performance.

## 3 Conclusions

In summary, in this work, we use a versatile, scalable, and facile solution combustion synthesis route to synthesize Fe-doped NiO_*x*_ nanoparticles, with average particle sizes ranging from 2.5 to 37 nm. We find that the electrochemical activity of the smallest sulfate-derived particles is more than 2 orders of magnitude larger than the largest nitrate-derived particles. The increase in activity cannot solely be accounted for by the ∼15 times increase in surface area due to the smaller particle size. To investigate the physical origin of the specific surface area-normalized activity, we use time-resolved optical spectroscopy. At water oxidation potentials, we detect the formation of oxidized nickel centers and find that the number of these species per specific surface area increases with the decreasing particle size, possibly due to the larger exposure of surface nickel sites. We also find that the water oxidation current exhibits a second-order dependence on the density of the oxidized Ni species. However, the kinetics of the reaction vary significantly with the nanoparticle size, being 2 orders of magnitude lower for the smallest sulfate-derived nanoparticles, attributed primarily to a stronger binding of *O species (i.e. a cathodic shift of species energetics). Our results thus suggest that the higher OER activity per geometric area of the smaller particles can be attributed to the larger charge accumulated per surface area, considering the slower reaction kinetics per oxidized state. Further improvements in the catalytic activities of materials that rely on cooperative effects between active states for water oxidation should investigate how to increase the density of active states per surface area, while also facilitating their interaction.

## 4 Methods

### 4.1 Synthesis

The different Fe_0.1_Ni_0.9_O samples (nitrate, chloride, sulfate, and acac) were synthesized using sol–gel autocombustion synthesis following a similar reported procedure.^12^ The four materials were synthesized by preparing separately the reactive mixtures of iron (0.1 molar part) and nickel (0.9 molar part) salts dissolved in appropriate solvents to form four distinct solutions. For the iron chloride, nitrate, and sulfate salts, a water/ethanol mixture was used, while for iron acetylacetonate (acac), acetone served as the solvent. The nickel nitrate (II) salts were dissolved in water/ethanol, with the final concentration in the mixture of 0.3 M. To the combined solution, ethylene glycol, acting as the fuel, was introduced to each solution in a 1:1 oxidant-to-fuel molar ratio. The pH value of the solution was initially adjusted to 1 using a diluted acid (hydrochloric, nitric, or sulfuric, depending on the corresponding iron salt). Solutions were thoroughly mixed by using magnetic stirring to ensure homogeneity. Then, ammonia was incrementally added to each solution to neutralize the pH to 7 and constantly monitored with a pH meter to ensure precision. The neutralized solutions were then left to stand at 90 °C for an extended period (16 h) to facilitate gel formation. Subsequently, the gels were dried at 120 °C for 6 h to eliminate excess water, ensuring the integrity of the gel structure. A small fraction (1–3 mg) of the resultant material was separated for subsequent thermal analysis to characterize the decomposition and combustion properties. The remaining bulk material was placed in a muffle furnace and subjected to autocombustion at 350 °C for 1 h to achieve the final Fe_0.1_Ni_0.9_O composition.

## 5 Materials Characterization

### 5.1 Scanning Electron Microscopy

SEM images were acquired by using a LEO GEMINI 1525 microscope. A 1.5 keV electron beam and secondary electron detector were used. The sample was covered with a conductive chromium coating of 10 nm.

### 5.2 X-Ray Photoelectron Spectroscopy

XPS measurements were performed using a Thermo Scientific K-alpha+ instrument at a base pressure of 2 × 10^–9^ mbar. Monochromated and microfocused Al Kα (*h*ν = 1486.6 eV) radiation was used. The ejected photoelectrons were analyzed by using an 180° double-focusing hemispherical analyzer with a two-dimensional detector.

### 5.3 Electrode Preparation

A portion of 5 mg of the as-prepared catalyst was dispersed in 1 mL of a solution of 987 μL of EtOH/H_2_O in a 3:1 ratio and 12.7 μL of FAA Fumatech anionomer (10% w/w with respect to the catalyst). An FTO glass slide was cleaned (sonicated in concentrated HCl, ethanol, and acetone for 10 min each) and covered with a Kapton tape, leaving an exposed area of 10 × 10 mm^2^. Four coatings of the catalyst ink, 125 μL each, were sprayed onto the FTO slide with an airbrush. The FTO slide was placed on a hot plate at 75 °C for the whole process.

### 5.4 Electrochemistry

Electrochemical measurements were performed using an Autolab potentiostat (PGSTAT 101). A three-electrode cell was used with a Pt mesh as the counter electrode and Ag/AgCl (saturated KCl) as the reference electrode. The standard potential of the reference electrode was calibrated against a reversible hydrogen electrode (constructed using a clean Pt wire immersed in an electrolyte and saturated with hydrogen gas). Electrochemical impedance spectroscopy measurements were used to determine the fitted uncompensated series resistance at OER-relevant potentials from 0.1 MHz to 1 Hz. The electrolyte used for all measurements was Fe-free 0.1 M KOH (Suprapur 95% KOH, Merck, Germany). Electrolyte purification was performed using the protocol described in ref (5). A 1 M solution of KOH was purified and diluted to 0.1 M for the electrochemical measurements. Two grams of 99.999% Ni(NO_3_)_2_.6H_2_O was dissolved in ∼4 mL of deionized water in an acid-cleaned polypropylene centrifuge tube. To this was added 20 mL of 1 M KOH, which resulted in the precipitation of highly pure Ni(OH)_2_. After shaking the mixture and centrifuging, the supernatant was decanted. Ni(OH)_2_ underwent three similar washing cycles; for each washing cycle, ∼20 mL of DI water and ∼2 mL of 1 M KOH were added to the tube. In the final step, the centrifuge tube was filled with 50 mL of 1 M KOH; the solid was redispersed; and the solution was left to rest for 3 h. The mixture was centrifuged, and the purified KOH was decanted for use.

### 5.5 Spectroelectrochemistry

A spectroelectrochemical cell was fitted with a Cary 60 UV–vis spectrometer (Agilent Technologies). Measurements were made under potentiostatic conditions, with the spectra being collected after the current had stabilized. Stepped potential measurements require a potential jump (pump) and an optical probe, as described in ref (27). Upon the application of a potential, optical absorption was monitored using a 100 W tungsten lamp (Bentham IL1), with an Oriel cornerstone 130 monochromator. The transmitted light was filtered by using several band-pass and long-pass filters (Comar Optics) and detected by using a silicon photodiode (Hamamatsu S3071). The photons were converted to a voltage signal, which was then passed through an amplifier (Costronics) and recorded using an oscilloscope (Tektronics TDS 2021c) and with a DAQ card (National Instruments, NI USB06211). The potentiostat used was PalmSens3. A home-built LabView software was used to acquire all optical and electrochemical data.

For open-circuit decay measurements, samples were deposited on ∼1 cm × 1 cm area of FTO substrates. Measurements were made in a three-electrode cell using a custom-built optical spectroscopy setup. A stabilized 10 mW tungsten-halogen light source from Thorlabs (SLS201L) was used with a collimating add-on (SLS201C). The light emitted from the lamp was transmitted through the sample and collected by using a 1 cm diameter liquid light guide (Edmund optics). Light transmitted to the spectrograph was first columnated and refocused using two 5 cm planoconvex lenses (Edmund) in order to optimally match the optical components of the spectroscope (Kymera 193i, Andor) and CCD camera (iDus Du420A-BEX2-DD, Andor). The detector was maintained at −80 °C during the measurements to ensure a high signal-to-noise ratio. An Ivium Vertex potentiostat was used. Data acquisition was facilitated by a custom-built LabView software. Measurements were taken in potentiostatic mode. The equilibration time at each potential was 10 s. This was followed by the measurement of the optical spectra. At each potential, 30 averages of the spectra were taken (each spectral acquisition takes ∼30 ms) before moving to the next potential. Simultaneously, the current was measured at each potential using an Ivium Vertex potentiostat. The potential was measured with respect to a Ag/AgCl reference electrode (saturated KCl), which was calibrated versus a reversible hydrogen electrode. A Pt mesh was used as the counter electrode.

## Supporting Information Available

ICP OES, cross section SEM, TEM, and cyclic voltammograms of the samples; additional optical absorption spectra; and calculation of extinction coefficient and reaction kinetics (PDF)
