# Supplementary material for: Unraveling the Role of Particle Size and Nanostructuring on the Oxygen Evolution Activity of Fe-Doped NiO
Source: ACS Catal. 2024 Jul 17;14(15):11389–99. doi: 10.1021/acscatal.4c02329 (PMC11301624; doi:10.1021/acscatal.4c02329)
Supplement: Supplementary file 1 — cs4c02329_si_001.pdf [file cs4c02329_si_001.pdf]

Supplementary Information

Unravelling the role of particle size and nanostructuring on the oxygen evolution activity of Fe-doped NiO

Reshma R Rao[1, 2, 6 *], Alberto Bucci[3, 6], Sacha Corby[4], Benjamin Moss[4], Caiwu Liang[1], Aswin Gopakumar[3], Ifan E.L. Stephens[1], Julio Lloret-Fillol[3,5 *], James R. Durrant[4]

[1]Department of Materials, Royal School of Mines, Imperial College London, South Kensington Campus, London SW7 2AZ, U.K.

[2]Grantham Institute – Centre for Climate Change and the Environment, Imperial College London, South Kensington Campus, London SW7 2AZ, U.K.

[3]Institute of Chemical Research of Catalonia (ICIQ), The Barcelona Institute of Science and Technology, Avinguda Països Catalans 16, 43007 Tarragona, Spain

[4]Department of Chemistry, Centre for Processable Electronics, Imperial College London, London W12 0BZ, United Kingdom

[5]Catalan Institution for Research and Advanced Studies (ICREA), Passeig Lluıs Companys, 23, 08010, Barcelona, Spain

[6] These authors contributed equally

* Corresponding authors

Reshma R Rao – reshma.rao@imperial.ac.uk

Julio Lloret-Fillol - jlloret@iciq.es

Characterization

Table S1: Results of ICP-OES analysis of doped metal in the NiO in percentage.

**Sample**	**%Fe**
Sulfate	11
Nitrate	10
Chloride	9
Acac	10

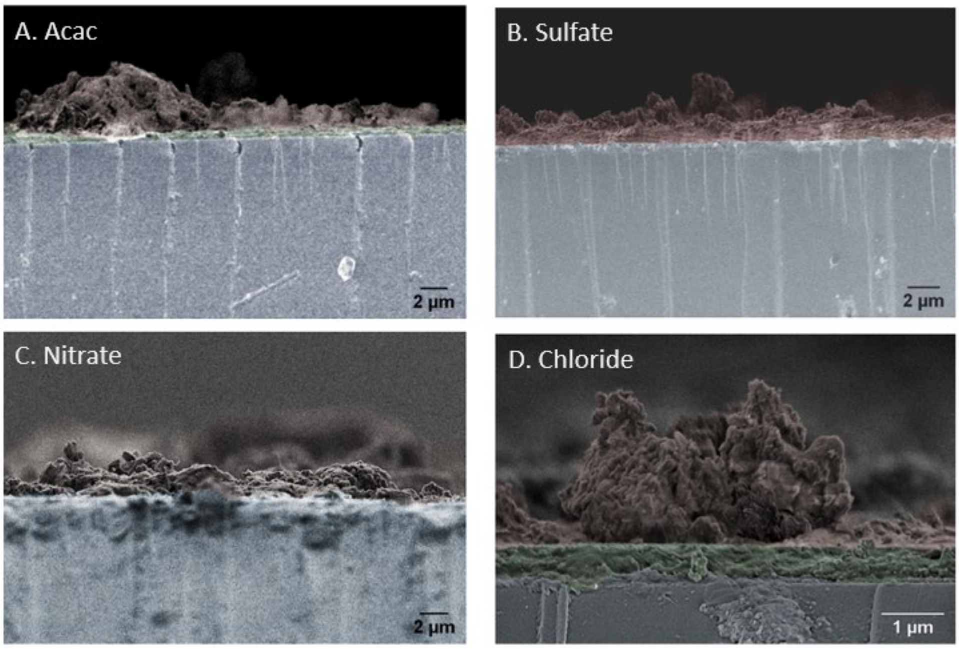

Figure S1: Cross section SEM images of the as-prepared (A) Acac- (B) Sulfate- (C) Nitrate- and (D) Chloride- derived $Fe_{0.1}Ni_{0.9}O$ samples. All the samples exhibit similar foamy morphology

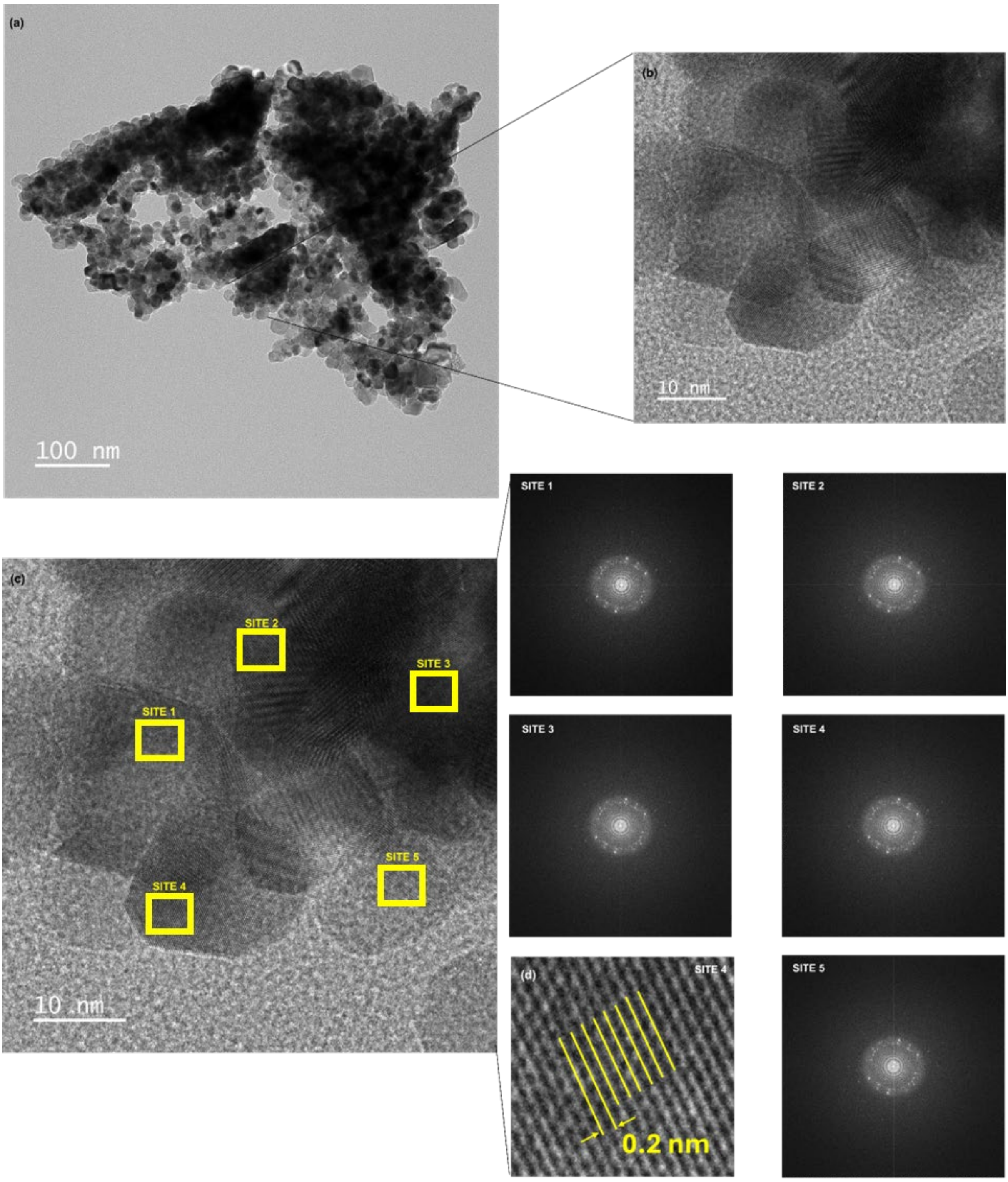

Figure S2: (A) HRTEM image of the NiO sample and its (B and C) magnified portions. FFT diffractograms from five different sites with (D) magnified site 4 (representative) showing the fringe-width of 0.2 nm matching with the pure NiO cubic crystal.

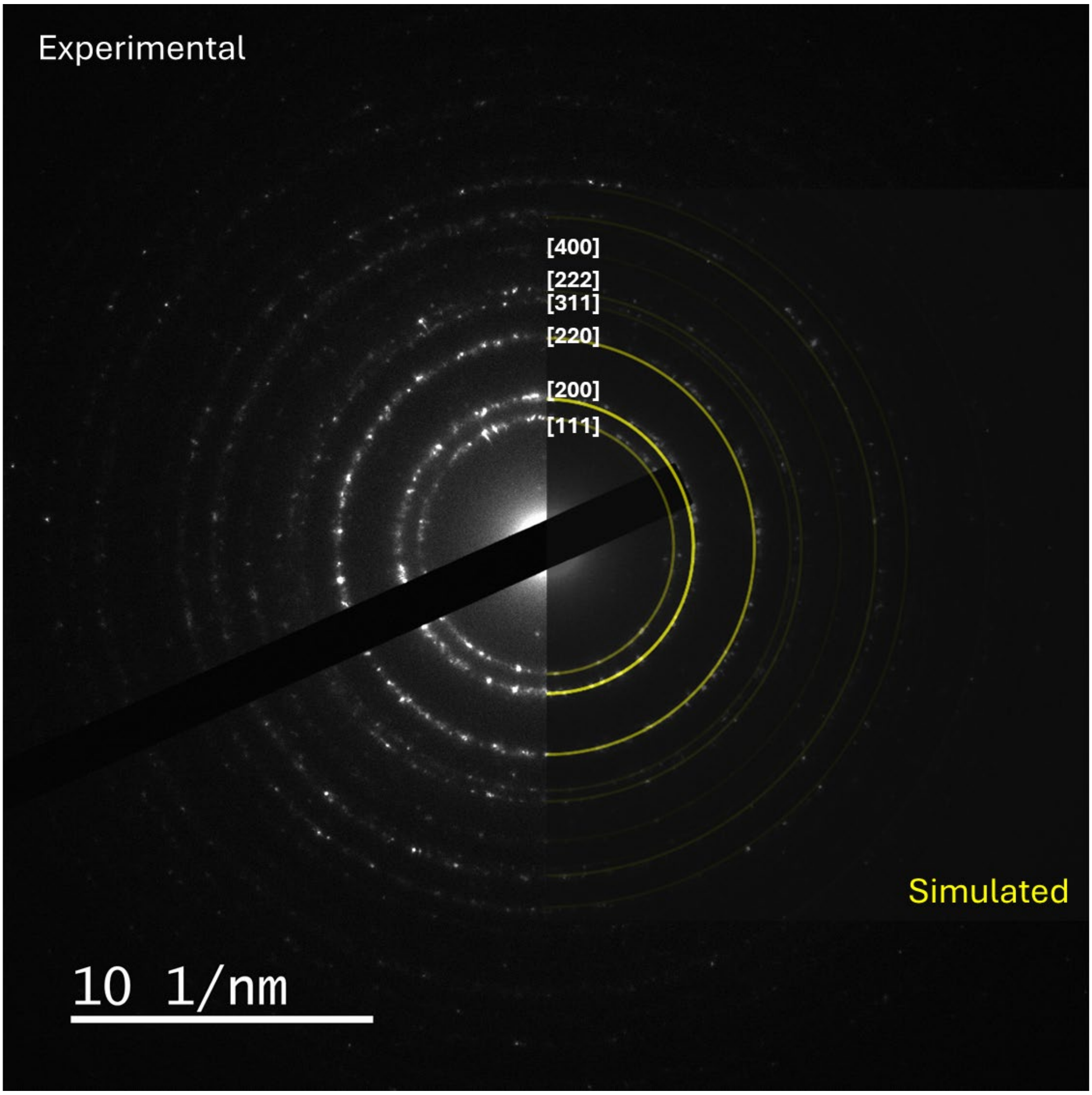

Figure S3: Selected area electron diffraction (SAED) ring pattern of the NiO sample with overlaid simulated pattern of pure NiO crystal (cubic).

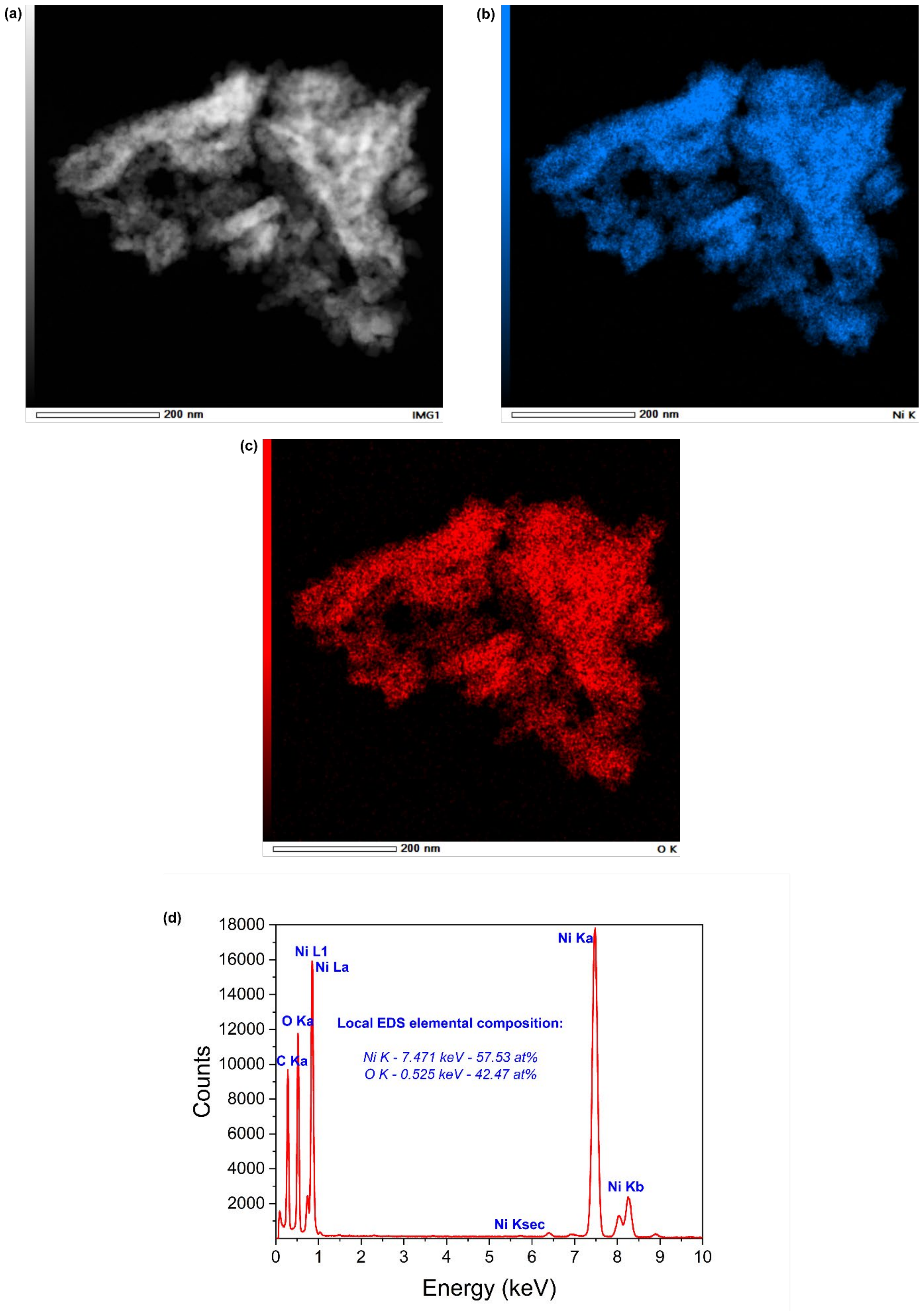

Figure S4: (A) STEM image with EDS maps of NiO sample showing the localized presence of (B) Ni (blue) and (C) O (red) and (D) local EDS spectra.

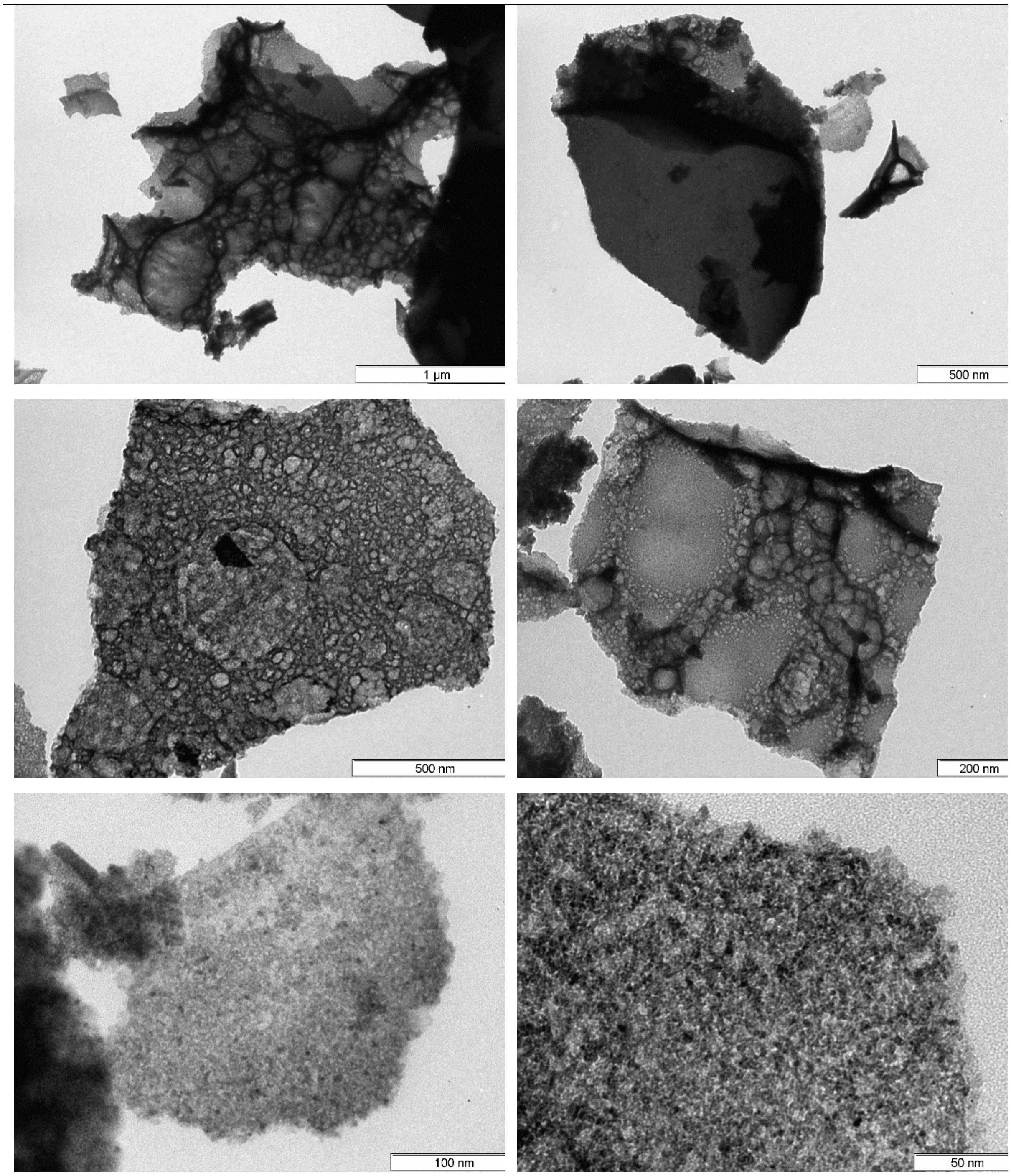

Figure S5: TEM images of the $Fe_{0.1}Ni_{0.9}O$ sulfate sample.

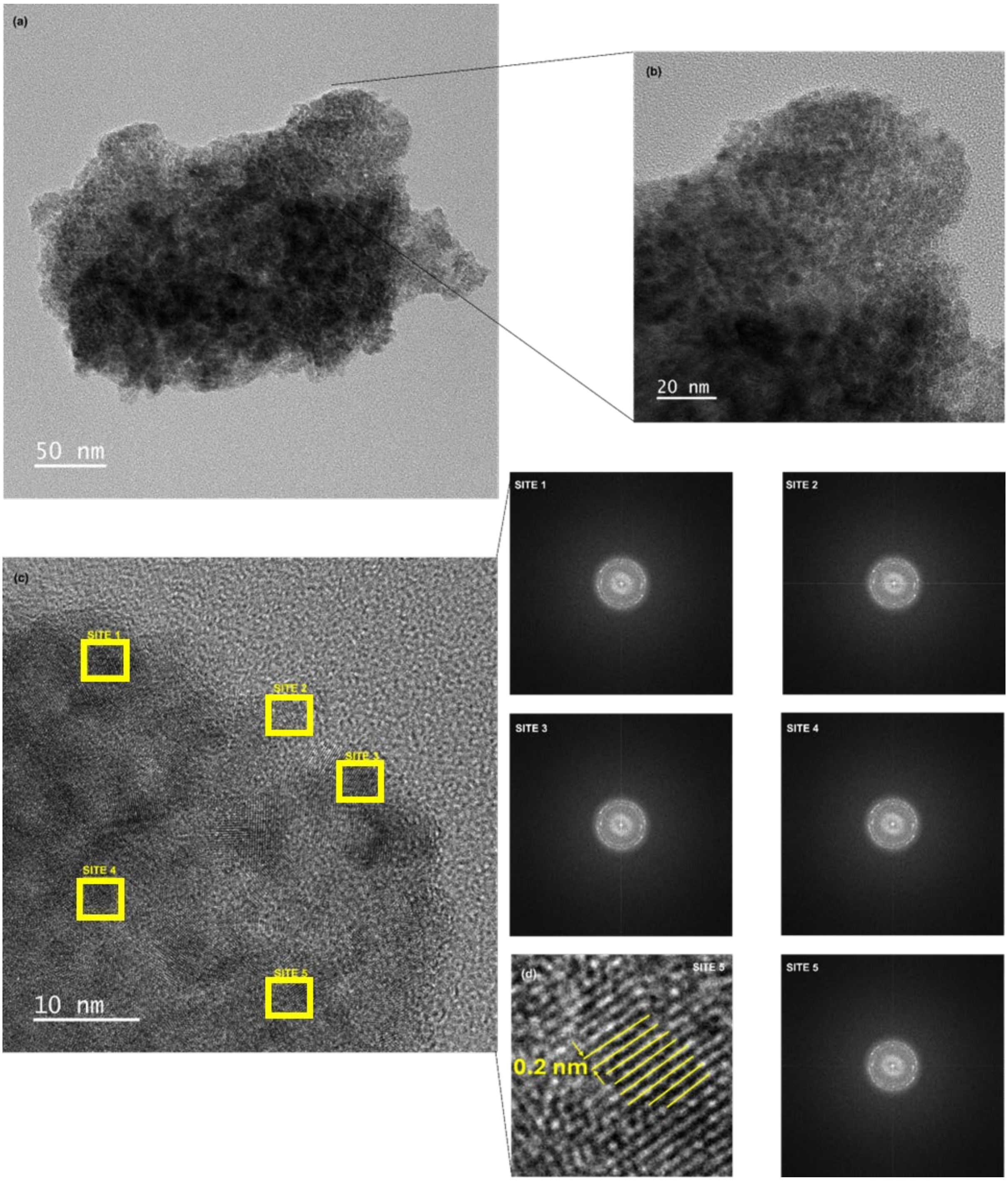

Figure S6: (A) HRTEM image of the $Fe_{0.1}Ni_{0.9}O$ sulfate sample and its (B and C) magnified portions. FFT diffractograms from five different sites with (D) magnified site 5 (representative) showing the fringe-width of 0.2 nm matching with the pure NiO cubic crystal.

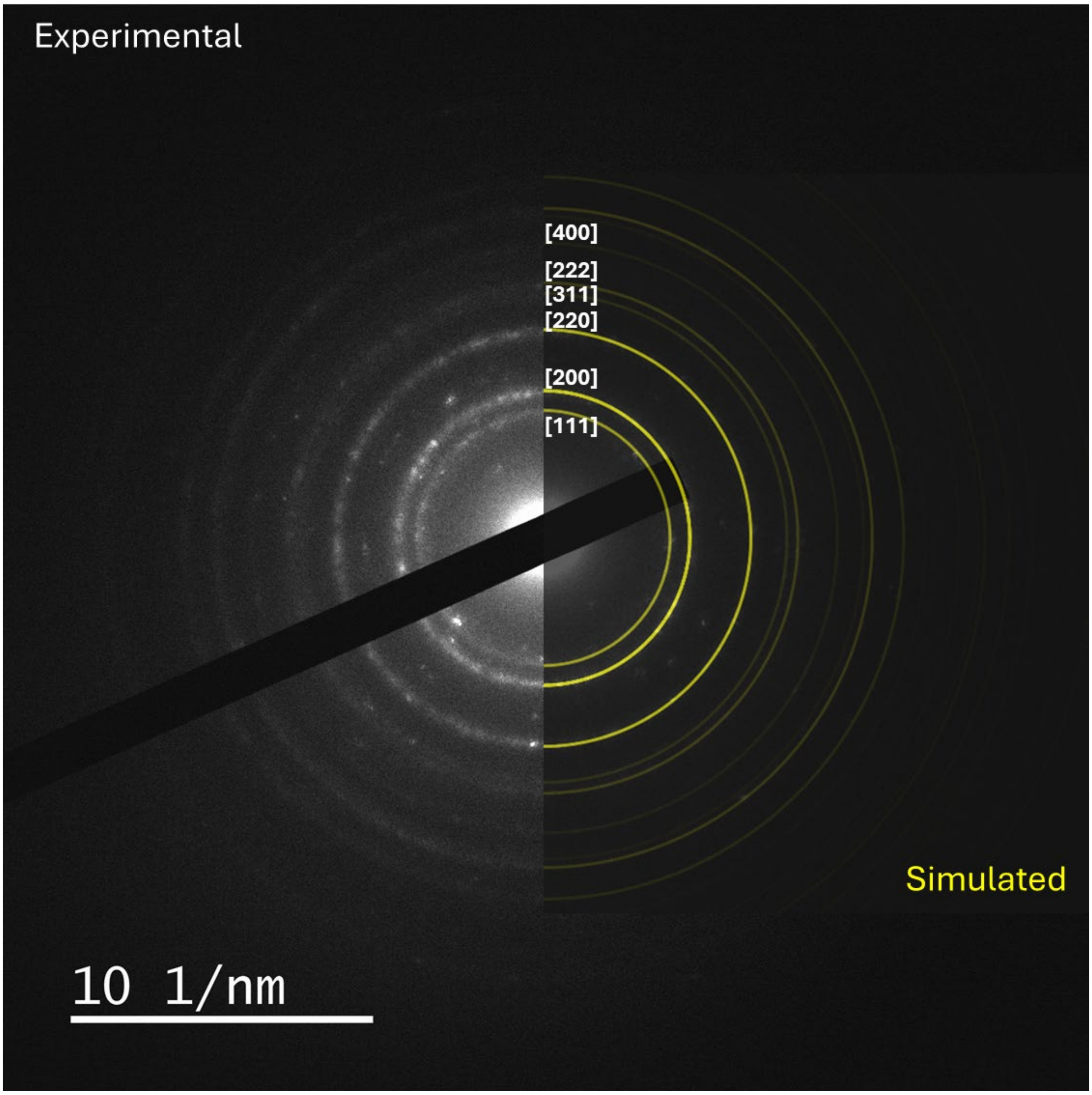

Figure S7: Selected area electron diffraction (SAED) ring pattern of the $Fe_{0.1}Ni_{0.9}O$ sulfate sample with overlaid simulated pattern of pure NiO crystal (cubic). Additional reflections indicate the presence of dopants (here, Fe). The occurrence of diffused rings denotes very small particles, resulting in the broadening of peaks in the powder XRD diffractograms as shown in Figure 1E in the main text.

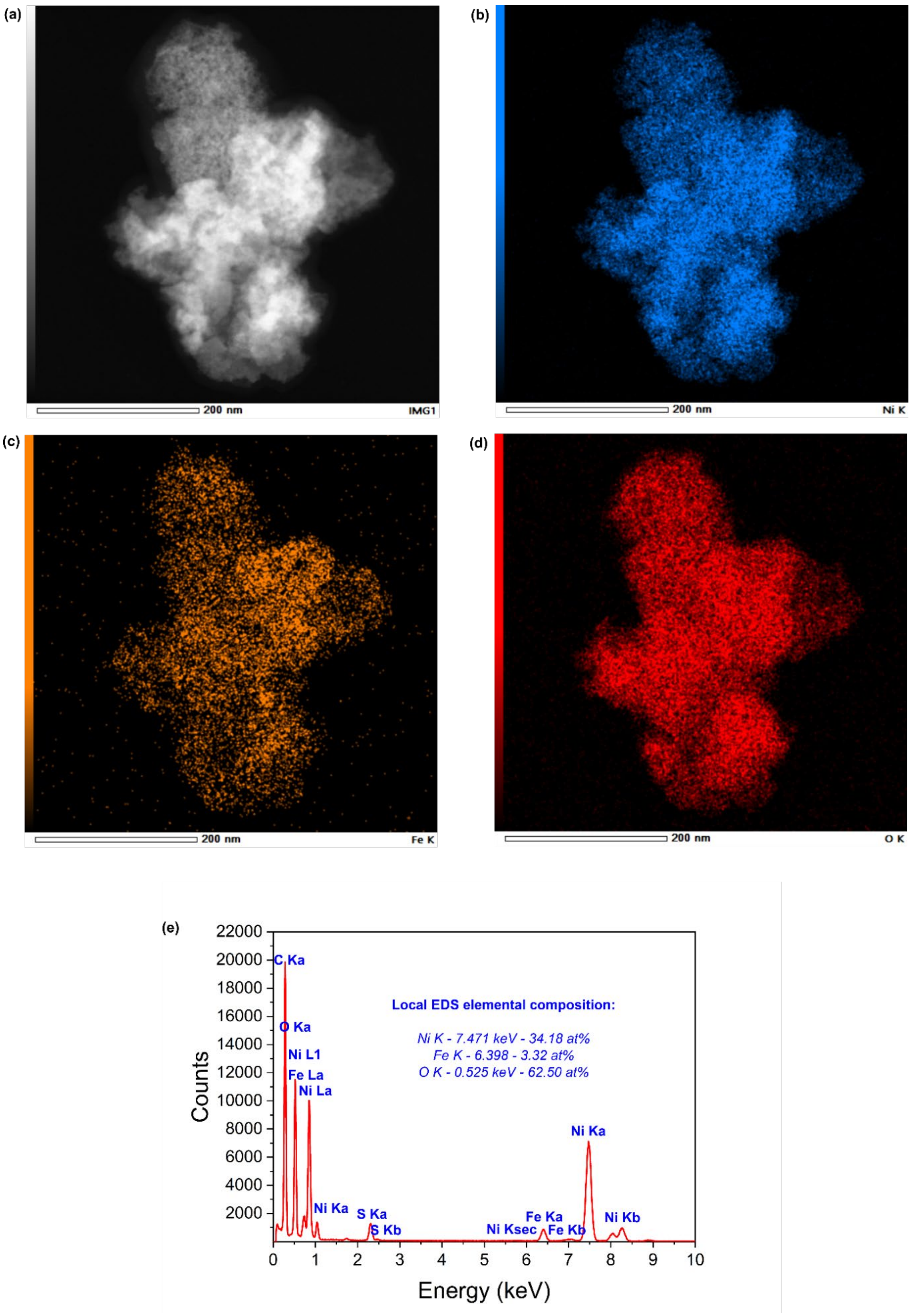

Figure S8: (A) STEM image with EDS maps of $Fe_{0.1}Ni_{0.9}O$ sulfate sample showing the localized presence of (B) Ni (blue), (C Fe (orange), (D) O (red) and (E) local EDS spectra. S trances are detected in the bulk.

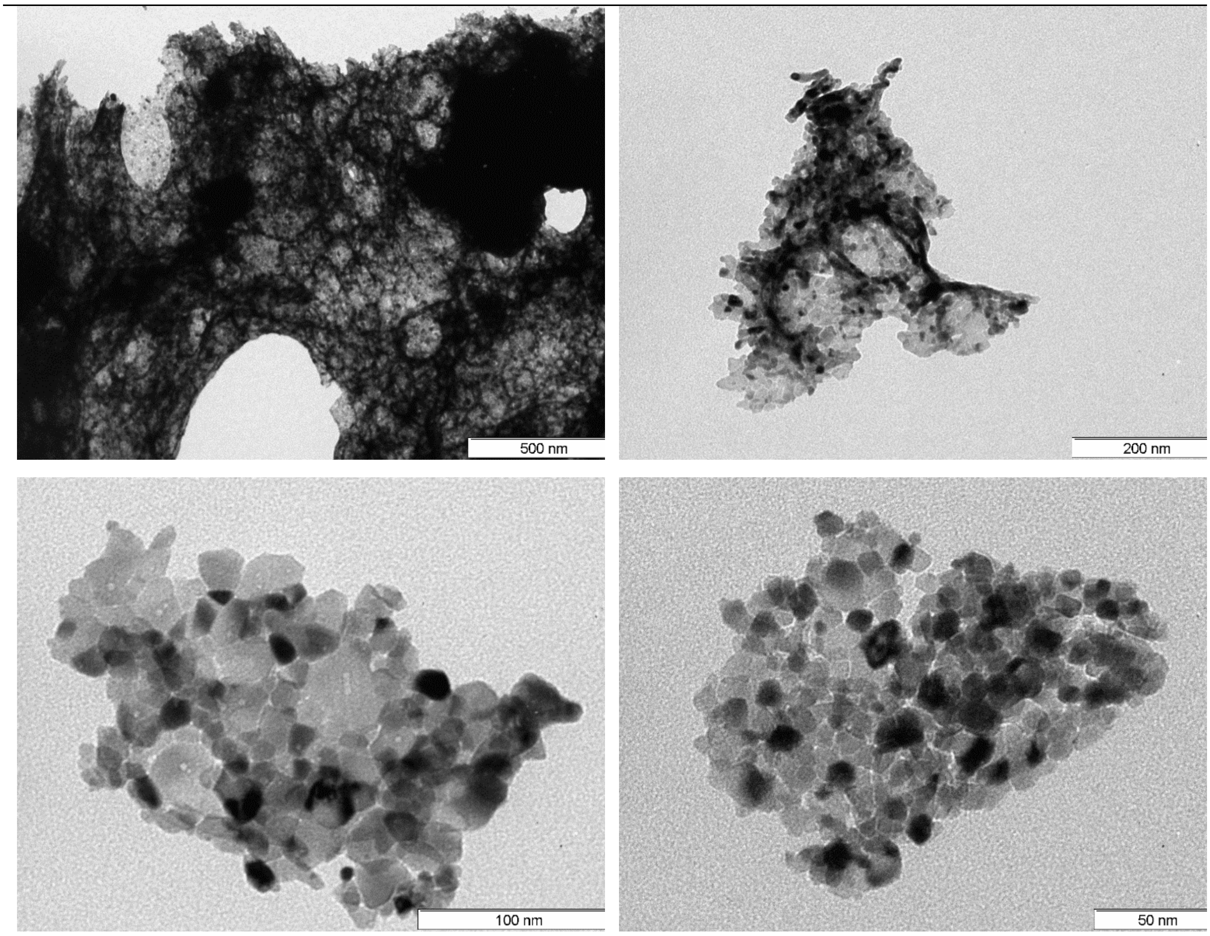

Figure S9: TEM images of the $Fe_{0.1}Ni_{0.9}O$ acac sample.

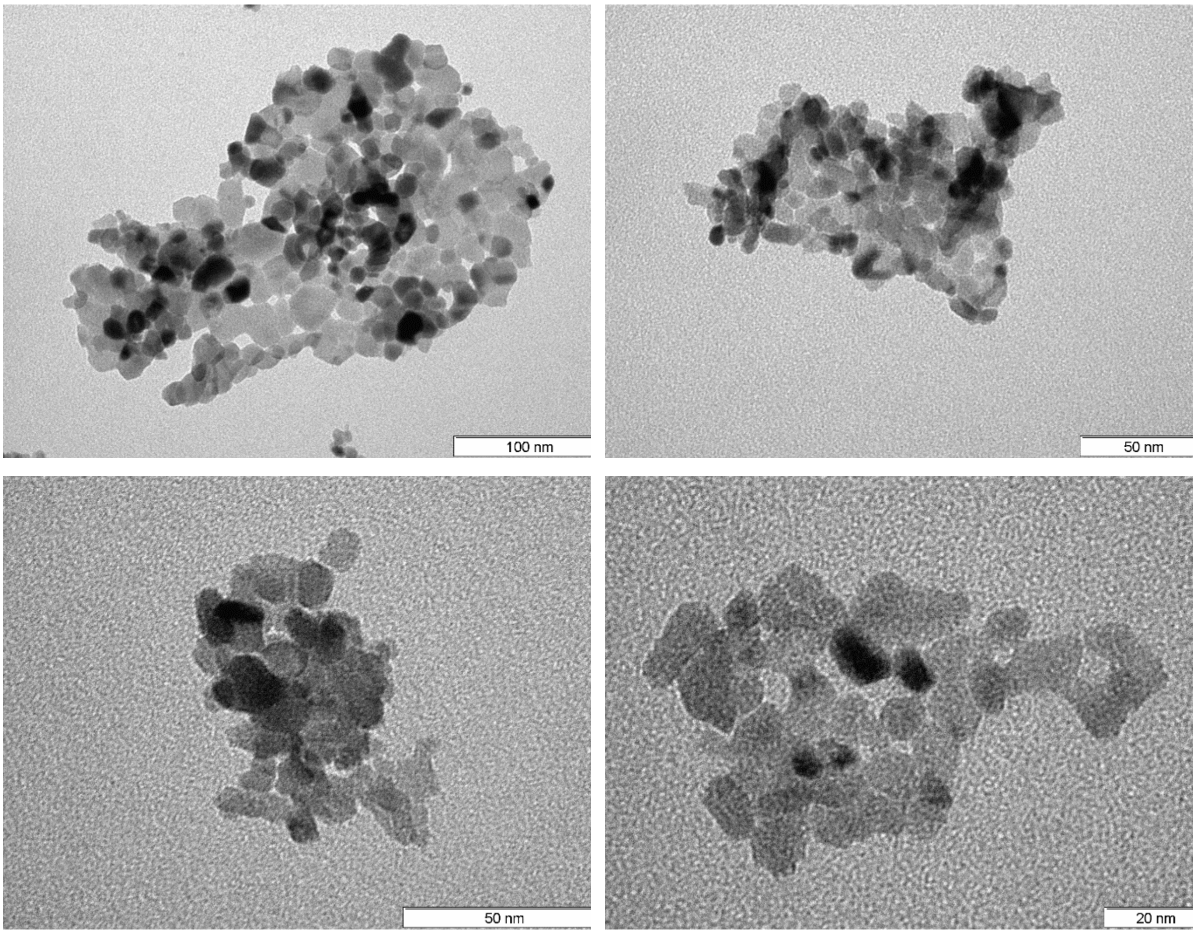

Figure S10: TEM images of the $Fe_{0.1}Ni_{0.9}O$ nitrate sample.

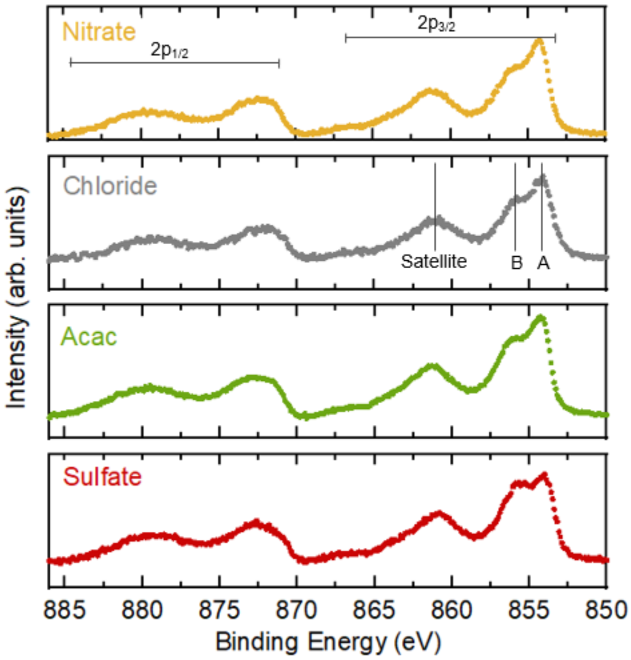

Figure S11: Ni *2p* XPS spectra of the as-prepared nitrate- (yellow), chloride- (grey), acac- (green) and sulfate- (red) $Fe_{0.1}Ni_{0.9}O$ samples. All samples show the presence of Ni^{3+} species.

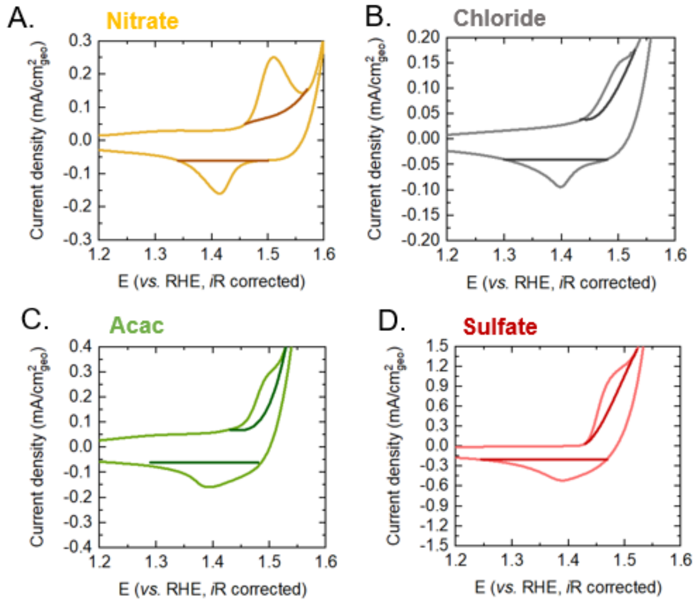

Figure S12: Cyclic voltammogram of the (A) nitrate – (B) chloride – (C) acac – and (D) sulfate – derived $Fe_{0.1}Ni_{0.9}O$ samples. All measurements were made in 0.1 M Fe- free KOH at a scan rate of 10 mV/s. The lines in darker shades of the corresponding colour indicate the baselines used to determine the area and redox peak centre of the anodic and cathodic redox transitions.

Spectroelectrochemistry

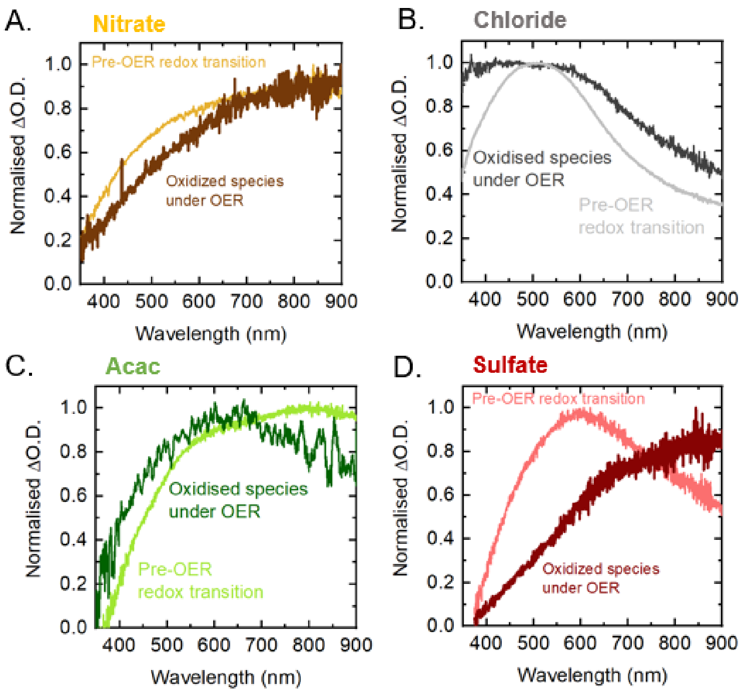

Figure S13: Normalized differential absorption for the species corresponding to the pre-OER redox transition (light) and oxidized species present at OER potentials (dark) for the (A) nitrate- (B) chloride- (C) acac- and (D) sulfate- derived $Fe_{0.1}Ni_{0.9}O$ samples.

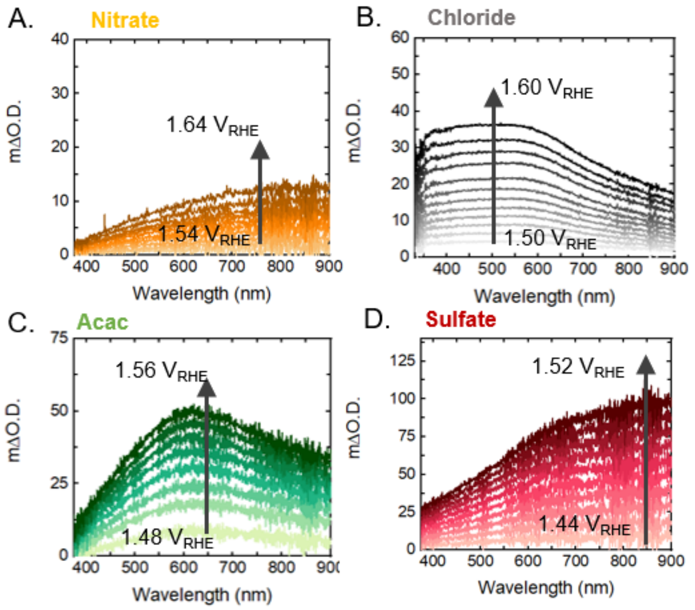

Figure S14: Differential UV-vis spectra as a function of potential (noted in the figure) for the (A) nitrate- (B) chloride- (C) acac- and (D) sulfate- derived $Fe_{0.1}Ni_{0.9}O$ samples.

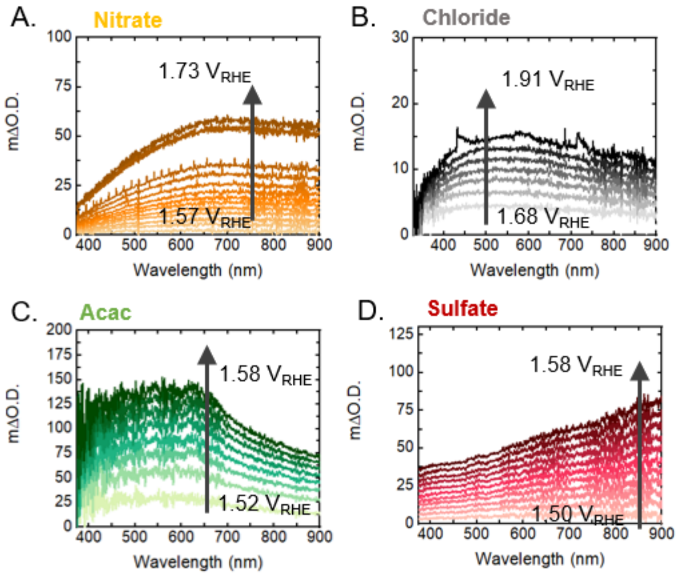

Figure S15: Differential UV-vis spectra as a function of potential (noted in the figure) for the (A) nitrate- (B) chloride- (C) acac- and (D) sulfate- derived $Zn_{0.1}Ni_{0.9}O$ samples.

Calculation of extinction coefficient

The extinction coefficient was calculated using the stepped potential spectroelectrochemistry method. A voltage pulse was applied, and the corresponding change in optical absorption and current density were measured simultaneously. The measured optical data is proportional to the density of oxidized species in the sample. Upon switching the potential back to the lower value, a reductive spike in the current is observed, which corresponds to the reduction in the oxidized species. The current-time response during the reductive spike can be integrated to deduce the charge corresponding to reduction in the oxidized states. Using the Lambert-Beer Law, the extinction coefficient can be extracted by plotting the optical signals as a function of the charge. The slope of the graph yields the extinction coefficient.

Beer-Lambert Law:

$$A = \varepsilon * c$$

where:

A = absorbance at a given wavelength,
ε = extinction coefficient
c = concentration of electrons per cm^2

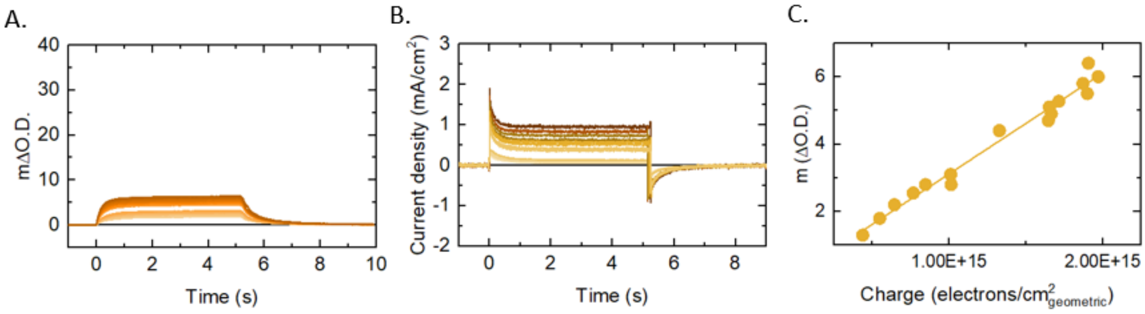

Figure S16: Calculation for nitrate-derived $Fe_{0.1}Ni_{0.9}O$. (a) electrochemical data and (b) optical data obtained from stepped voltage absorption spectroscopy for increasingly larger potential steps in the OER region. (c) Extinction coefficient obtained from gradient of optical signal to charge. The potential steps were from 1.58 V_{RHE} to 1.66 V_{RHE}. Measurements were made at a wavelength of 750 nm in Fe-free 0.1 M KOH.

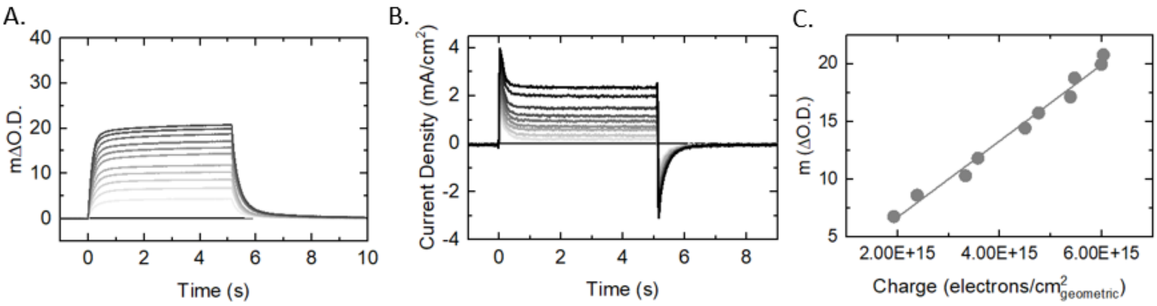

Figure S17: Calculation for chloride-derived $Fe_{0.1}Ni_{0.9}O$. (a) electrochemical data and (b) optical data obtained from stepped voltage absorption spectroscopy for increasingly larger potential steps in the OER region. (c) Extinction coefficient obtained from gradient of optical signal to charge. The potential steps were from 1.57 V_{RHE} to 1.63 V_{RHE}. Measurements were made at a wavelength of 500 nm in Fe-free 0.1 M KOH.

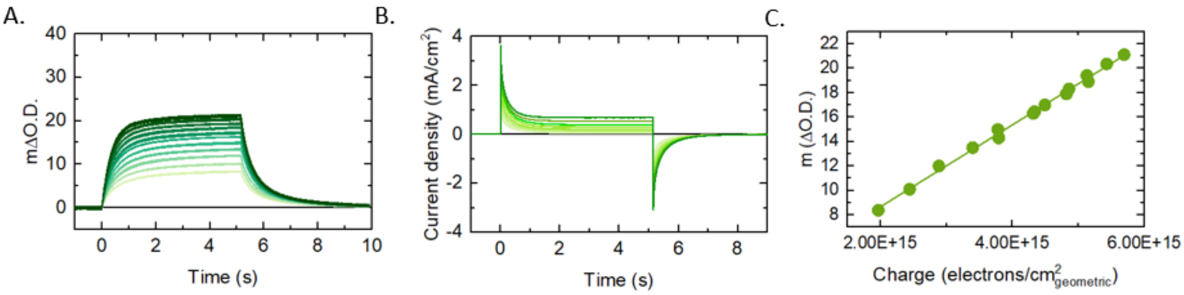

Figure S18: Calculation for acac-derived $Fe_{0.1}Ni_{0.9}O$. (a) electrochemical data and (b) optical data obtained from stepped voltage absorption spectroscopy for increasingly larger potential steps in the OER region. (c) Extinction coefficient obtained from gradient of optical signal to charge. The potential steps were from 1.50 V_{RHE} to 1.55 V_{RHE}. Measurements were made at a wavelength of 650 nm in Fe-free 0.1 M KOH.

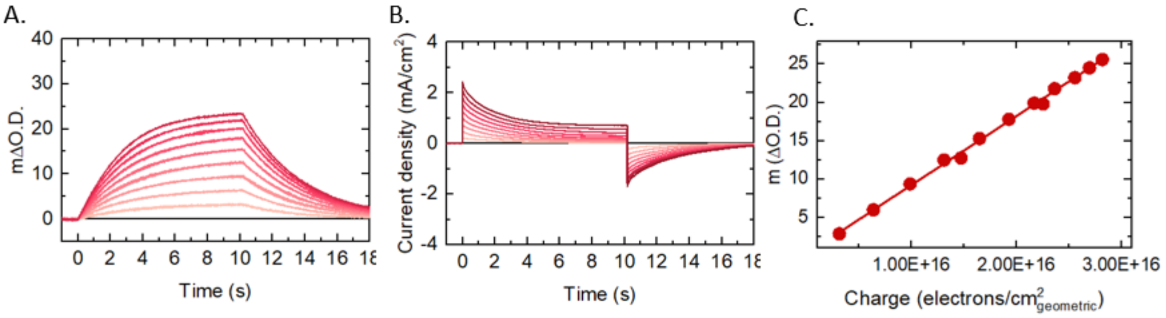

Figure S19: Calculation for sulfate-derived $Fe_{0.1}Ni_{0.9}O$. (a) electrochemical data and (b) optical data obtained from stepped voltage absorption spectroscopy for increasingly larger potential steps in the OER region. (c) Extinction coefficient obtained from gradient of optical signal to charge. The potential steps were from 1.485 V_{RHE} to 1.51 V_{RHE}. Measurements were made at a wavelength of 850 nm in Fe-free 0.1 M KOH.

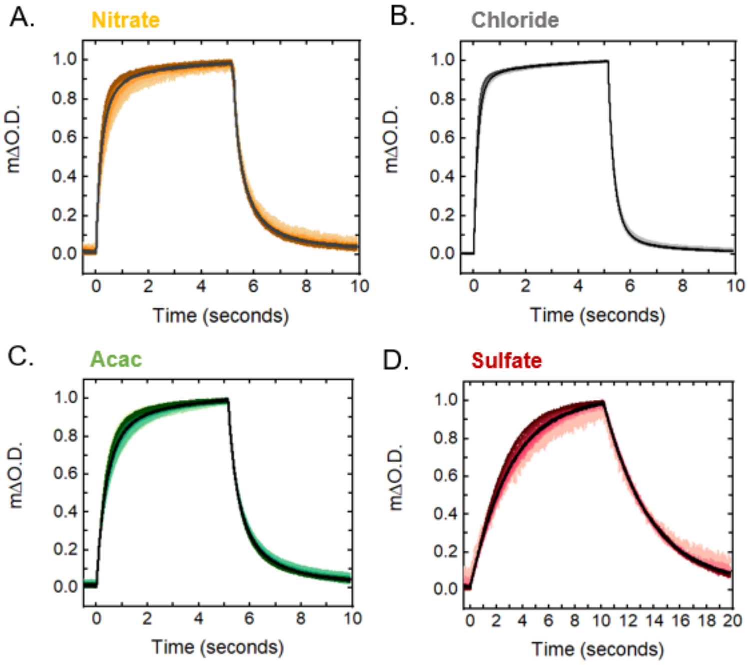

Figure S20: Normalized absorption data for the stepped potential measurements shown in Figure S16-S19 for the (A) nitrate- (wavelength = 750 nm) (B) chloride- (wavelength = 500 nm) (C) acac- (wavelength = 650 nm) and (D) sulfate- (wavelength = 850 nm) derived $Fe_{0.1}Ni_{0.9}O$ samples. In each panel, a gradient of the corresponding color has been used to represent the data, where the lightest colour represents the lowest potential and the darkest colour of the gradient represents the largest potential step in the measurement. The black curves show the average of all the data sets.

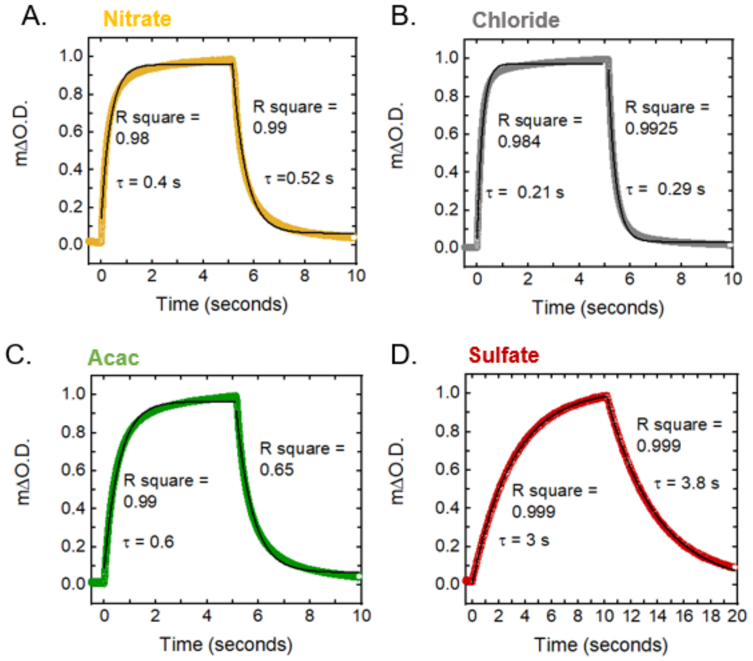

Figure S21: Normalized average absorption for the stepped potential measurements for (A) nitrate- (wavelength = 750 nm) (B) chloride- (wavelength = 500 nm) (C) acac- (wavelength = 650 nm) and (D) sulfate- (wavelength = 850 nm) derived $Fe_{0.1}Ni_{0.9}O$ samples. The points represent the averaged data and the lines represent the best fit based on a single exponential model. The time constant for oxidation or reduction of these species has been annotated.

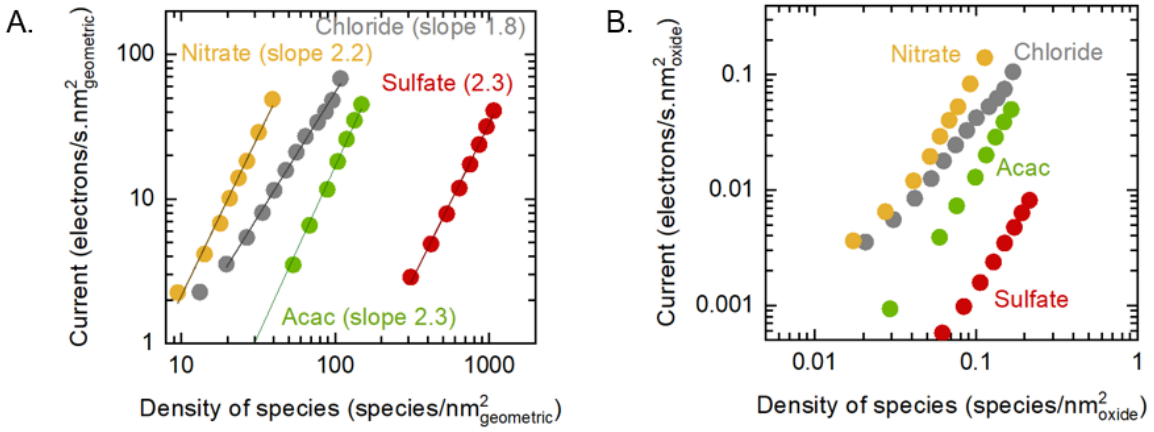

Figure S22: Log–log plot of the current density as a function of the density of oxidized species normalised to the (A) geometric area of the electrode and (B) oxide surface area.

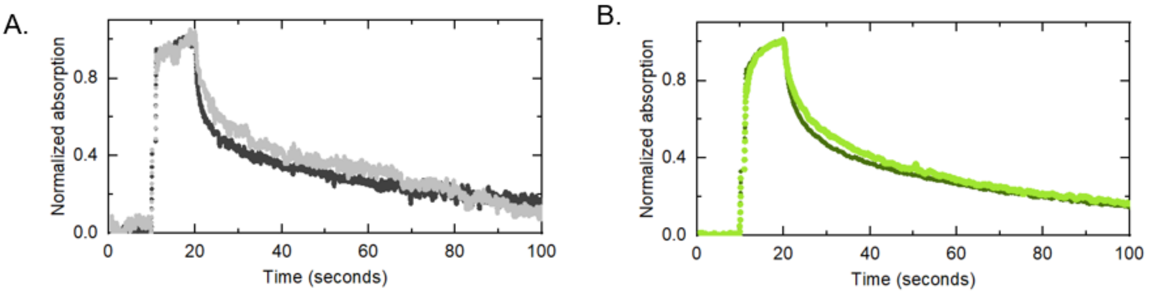

Figure S23: Open Circuit decay measurements for the (A) chloride derived sample from 1.57 V_{RHE} (~ 0.06 species/nm^2_{oxide}), grey and 1.59 V_{RHE} (~ 0.08 species/nm^2_{oxide}), black. The sample is held at constant potential from 10 to 20 seconds followed by open circuit decay from 20 to 100 seconds. Data is reported for a wavelength of 500 nm. (B) acac derived sample from 1.51 V_{RHE} (~ 0.039 species/nm^2_{oxide}), light green and 1.53 V_{RHE} (~ 0.05 species/nm^2_{oxide}), dark green. The sample is held at constant potential from 20 to 50 seconds followed by open circuit decay from 50 to 100 seconds. Data is reported for a wavelength of 650 nm.
